# Paired plant immune CHS3-CSA1 receptor alleles form distinct hetero-oligomeric complexes

**DOI:** 10.1126/science.adk3468

**Published:** 2024-02-16

**Authors:** Yu Yang, Oliver J. Furzer, Eleanor P. Fensterle, Shu Lin, Zhiyu Zheng, Nak Hyun Kim, Li Wan, Jeffery L. Dangl

**Affiliations:** 1 Department of Biology, University of North Carolina at Chapel Hill, Chapel Hill, NC, USA; 2 Howard Hughes Medical Institute, University of North Carolina at Chapel Hill, Chapel Hill, NC, USA; 3 National Key Laboratory of Plant Molecular Genetics, CAS Center for Excellence in Molecular Plant Sciences, Institute of Plant Physiology and Ecology, Chinese Academy of Sciences, Shanghai, China

## Abstract

Plant intracellular nucleotide-binding leucine-rich repeat receptors (NLRs) analyzed to date oligomerize and form resistosomes upon activation to initiate immune responses. Some NLRs are encoded in tightly linked co-regulated head-to-head genes whose products function together as pairs. We uncover the oligomerization requirements for different *Arabidopsis* paired CHS3-CSA1 alleles. These pairs form resting-state heterodimers that oligomerize into complexes distinct from NLRs analyzed previously. Oligomerization requires both conserved and allele-specific features of the respective CHS3 and CSA1 Toll-like interleukin-1 receptor (TIR) domains. The receptor kinases BAK1 and BIRs inhibit CHS3-CSA1 pair oligomerization to maintain the CHS3-CSA1 heterodimer in an inactive state. Our study reveals that paired NLRs hetero-oligomerize and likely form a distinctive “dimer of heterodimers” and that structural heterogeneity is expected even among alleles of closely related paired NLRs.

## Abstract (summary)

**INTRODUCTION:**

Plants rely on an innate immune system to defend themselves against pathogen invasion. Nucleotide-binding (NB) leucine-rich repeat (LRR) receptors (NLRs) are important components of intracellular innate immunity across all kingdoms. Plant NLRs are classified into two major groups, CNLs and TNLs, based on the presence of different N-terminal domains: Most NLRs express either a coiled-coil (CC) domain (CNLs) or a Toll-like interleukin-1 receptor (TIR) domain (TNLs). Some NLRs function as singletons, with one NLR protein mediating both pathogen recognition and subsequent downstream immune activation. However, some NLRs are encoded in a head-to-head orientation on the chromosome and function together—these are called paired NLRs. One member of each paired NLR is a so-called sensor that recognizes pathogen proteins or their activities and then activates an associated executor NLR to initiate immune signaling. Ligand-induced formation of various types of NLR oligomers, called resistosomes, mediates NLR function. However, oligomerization status and resistosome formation of paired NLRs are not currently understood.

**RATIONALE:**

*NLRs* are the most variable gene families in plants. Our recent studies demonstrated that allelic *Arabidopsis* TNL CHS3-CSA1 pairs can be divided into three phylogenetic clades. CHS3 is the sensor, and CSA1 is the executor. The CHS3-CSA1 TNL alleles evolved two different types of pairs and two separable regulatory modes for their activation across these three clades. We deployed two functionally distinct CHS3-CSA1 allelic pairs in a model to study the diverse oligomerization requirements and specific mechanism of activation of this paired TNL family.

**RESULTS:**

We found that the sensor CHS3 and executor CSA1 from clade 2 or clade 3 form the same size oligomers only in combination with their genetically encoded partners. Selfassociation of either CHS3 or CSA1 is weak in the absence of its partner, but heterocomplexes of CHS3 and CSA1 are readily detected when they are coexpressed. CHS3 and CSA1 from clade 2 or clade 3 oligomerize and form hetero-oligomers but not separate homooligomers. Mutations in the AE interface and the BB-loop of both CHS3 and CSA1 TIR domains suggested that the AE interface in the TIR domain of both CHS3 and CSA1 mediates essential head-to-head interaction for heterodimer formation and that the BB-loop in the TIR domains mediates essential head-to-tail interaction and dimerization of the heterodimers, eventually forming a heterotetramer. Mutational analysis of CHS3-CSA1 pairs from both clades also defined clade-specific TIR domain requirements for function and oligomerization, suggesting that TNL CHS3-CSA1 pairs from different clades form a distinct hetero-oligomeric complex. Coexpression of the previously defined negative regulators BAK1 or BIRs with the CHS3-CSA1 pair inhibited oligomerization but not heterodimer formation. These results suggest that paired NLRs exist at rest as heterodimers but form distinct hetero-oligomeric complexes to function and that the inactive state of paired NLRs can be maintained through the suppression of oligomerization by the negative regulators.

**CONCLUSION:**

A stepwise mechanism of paired TNL CHS3-CSA1 alleles immune activation is proposed on the basis of our findings and on the basis of previous biochemical studies. In the resting state, the majority of CHS3 and CSA1 from clades 2 or 3 form heterodimers and cannot further oligomerization because of the negative regulation by BAK1 and BIR proteins. Upon modulation of the BAK1 and/or BIRs by pathogens, host damage–associated patterns, or other mechanisms, suppression is relieved and the heterodimers of the CHS3-CSA1 pair further dimerize and likely form a distinct “dimer of heterodimers” oligomer. The interaction of CHS3 and CSA1 TIR domains leads to opening of the two CSA1 nicotinamide adenine dinucleotide hydrolase (NADase) active sites to initiate immune response and cell death. Our work uncovers the diverse oligomerization requirements of different CHS3-CSA1 alleles and suggests that structural heterogeneity can be expected even among alleles of closely related paired NLRs.

## Graphical Abstract

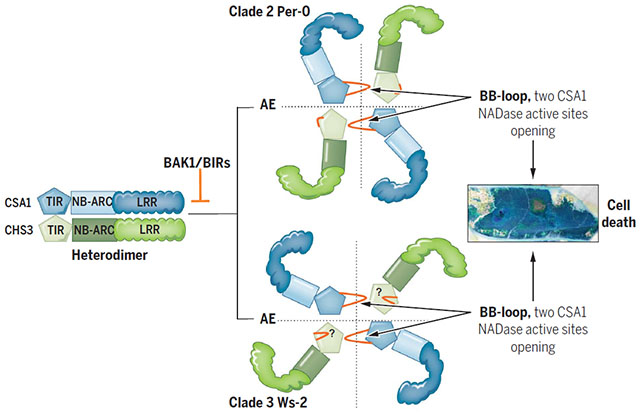

**The activation and distinct hetero-oligomer formation of paired NLRs.** The TNL CHS3 and CSA1 alleles form a resting state heterodimer, and this inactive state is maintained by the negative regulators BAK1 and BIRs. Upon modulation of BAK1 and/or BIRs activity, the heterodimers of the CHS3-CSA1 pair dimerize and likely form a distinct dimer of heterodimers oligomer. The interaction of TIR domains induces opening of two CSA1 NADase active sites, which are essential for CHS3-CSA1 pair–mediated cell death.

Plant intracellular nucleotide-binding leucine-rich repeat receptors (NLRs) play essential roles in innate immune systems. Some NLRs directly bind virulence factor effectors that are secreted and delivered into the plant cell by pathogens. Other NLRs indirectly detect host proteins modified by effectors (known as guardees or decoys of guardees). Either mode of NLR activation results in effector-trigged immunity (ETI) (1–6), usually accompanied by rapid calcium influx, a respiratory oxidative burst, transcriptional reprogramming, and cell death at the infection site that is referred to as the hypersensitive response (HR) (1–6). NLRs are broadly classified into three subgroups on the basis of their N-terminal domains. Their N termini typically contain Toll-like interleukin-1 receptor (TIR) domains, coiled-coil (CC) domains, or RPW8 (RESISTANCE TO POWDERY MILDEW 8)–like CC domains. NLRs carrying these domains are named TNLs (TIR-NLRs), CNLs (CC-NLRs), or RNLs (CC_R_-NLRs) (7–13), respectively.

NLRs can function as singletons, in genetically linked sensor-executor pairs (also known as paired NLRs or NLR pairs), or in genetically unlinked sensor-helper networks (14–17). Singleton NLRs include the *Arabidopsis* CNL ZAR1 (HOPZ-ACTIVATED RESISTANCE 1), which forms an oligomeric pentamer resistosome upon effector recognition. This resistosome relocates to the plasma membrane, where it acts as a cation channel to mediate Ca^2+^ influx (18–20). By contrast, many TNLs also function as sensor NLRs but require unlinked, ancient, and conserved helper RNLs to activate immune responses. The sensor TNLs RPP1 (RECOGNITION OF PERONOSPORA PARASITICA 1) and ROQ1 (RECOGNITION OF XOPQ 1) oligomerize to form tetrameric resistosomes upon activation (21, 22). TNL resistosomes are nicotinamide adenine dinucleotide hydrolases (NADases) that generate nucleotide-derived small signaling molecules (21–23). These induce the heterodimerization of either of two EDS1 heterodimers that in turn are required for the oligomerization and activation of downstream RNLs (24–29). RNLs, like CNLs, function as cation channels to mediate Ca^2+^ influx, defense, and cell death (24–29). Thus, both autonomous singleton CNLs and TNLs acting through RNLs end up signaling through resistosome-mediated calcium influx. Similarly, activation of a large class of sensor NLRs from a variety of solanaceous species enables the oligomerization of a limited array of differentially redundant helper NLRs called NRCs (17, 30, 31). NRC (NLR-REQUIRED for CELL DEATH) oligomers do not obviously contain sensor NLRs, which leads to an activation-release model for NLRs in the NRC immune receptor network (30–32).

In contrast to singletons and sensor-helper networks, paired *NLR* genes are encoded in head-to-head orientation, adjacent to one another. Such pairs make up ~10% of the NLR repertoire in *Arabidopsis* genomes (33). Each pair consists of a specialized “sensor” for effector perception and an “executor” for immune activation (34–44). To date, postactivation oligomerization and requirements for the oligomerization of paired NLRs are still elusive.

*Arabidopsis* allelic TNL CHS3-CSA1 (CHILLING SENSITIVE 3-CONSTITUTIVE SHADE-AVOIDANCE 1) pairs have been divided into three phylogenetic clades (33, 43) (fig. S1A). CHS3 is the proposed sensor NLR, and CSA1 is the proposed executor NLR in this pair (33, 43). The clade 1 sensor CHS3 proteins contain a putative effector-binding integrated decoy domain (ID) (44), which is lacking in clades 2 and 3 (33, 43) (fig. S1A). CHS3-CSA1 pairs in these three clades evolved two separable regulatory modes. One is mediated by the ID domain on clade 1 CHS3 sensors, whereas the other relies on CHS3-CSA1 pairs from all clades detecting the presence or perturbation of receptor-like kinase (RLK) proteins BAK1 (BRI1-ASSOCIATED RECEPTOR KINASE) and BIRs (BAK1-INTERACTING RECEPTOR-LIKE KINASEs) (43). In this work, we analyze the activation and oligomerization of different CHS3-CSA1 pairs from clades 2 and 3. We find that these TIR domain (TNL) pairs form heterodimers that oligomerize into distinct hetero-oligomeric complexes with common and clade-specific structural requirements. Moreover, BAK1 and BIRs act as negative regulators that inhibit the oligomerization—but not heterodimer formation—of the CHS3-CSA1 pair to maintain their inactive state. Kinase activity is not required for the negative regulatory function of these RLKs.

## CHS3-CSA1 TNL pairs oligomerize

We used blue native–polyacrylamide gel electrophoresis (BN-PAGE) to test oligomerization of CHS3-CSA1 TNL pairs from each of the three clades. We detected oligomerization of clade 2 and clade 3 CHS3-CSA1 pairs, as detailed below; therefore, we focused on them. Negative results for the clade 1 pair are not shown. Coexpression of paired CHS3 and CSA1 from clade 2 or clade 3 was sufficient to trigger strong hypersensitive-like cell death in *Nicotiana tabacum*, but neither CHS3 nor CSA1 expression alone was sufficient (43). This cell death phenotype was dependent on the conserved glutamic acid (E) in the CSA1 TIR domain and intact P-loops of both CHS3 and CSA1 (43). The conserved glutamic acid (E) in the CSA1 TIR domain is indispensable for NADase catalytic activity (43), and the P-loop typically conserved in NLR proteins is necessary for adenosine 5’-triphosphate (ATP) binding and NLR oligomerization (22–26). We recapitulated these findings using CHS3-CSA1 pairs from the clade 2 accession Per-0 and the clade 3 accession Ws-2 (fig. S1, A to C).

**Fig. 1.**
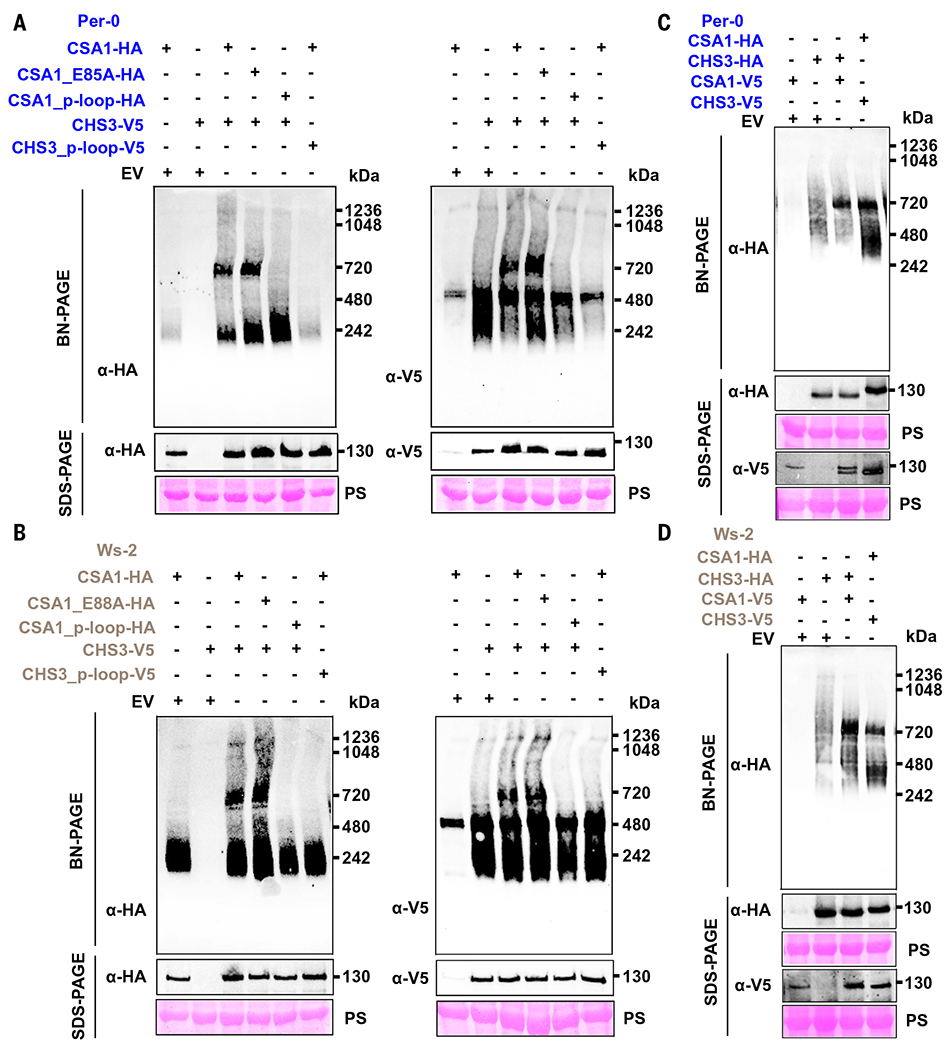
CSA1 and CHS3 oligomerize in a partner- and P-loop–dependent manner. (**A** and **B**) Both CSA1 and CHS3 from clade 2 Per-0 (blue) (A) or clade 3 Ws-2 (gray) (B) are capable of oligomerization dependent on their respective paired partner. Note that the clade 2 and clade 3 color schemes are maintained throughout. (Left) HA-tagged CSA1. (Right) V5-tagged CHS3. Amino acid abbreviations: E, glutamic acid; A, alanine. HA and V5 are the epitope tags. p-loop indicates P-loop dead mutant. PS (ponceau stain) indicates protein loading. (**C** and **D**) CSA1 and CHS3 from either clade 2 (C) or clade 3 (D) form similar size oligomers.

We then used wild-type CHS3-CSA1 pairs from clade 2 Per-0 or clade 3 Ws-2 and mutants derived from them to assay for oligomerization using BN-PAGE. We observed that oligomerization into a high–molecular weight band at apparent molecular mass of ~720 kDa was only detected when CHS3 and CSA1 were coexpressed and that both sensor CHS3 and executor CSA1 from clade 2 Per-0 or clade 3 Ws-2 were able to oligomerize (Fig. 1, A and B). We then explored whether the TIR catalytic residue of CSA1 and intact P-loops are required for CHS3 and CSA1 oligomerization. We found that the TIR catalytic activity of CSA1 was dispensable for oligomerization of CSA1 and CHS3 but that intact P-loops of CSA1 and CHS3 were required (Fig. 1, A and B).

Because the size of the CHS3 monomer (predicted molecular weight = ~125 kDa) is slightly smaller than that of CSA1 (predicted molecular weight = ~136 kDa) in both clade 2 Per-0 and clade 3 Ws-2, we swapped tags between CSA1 and CHS3 to compare the oligomer size. Coexpression of the new constructs resulted in similar cell death phenotypes (fig. S1, D and E). In addition, we used hemagglutinin (HA) antibody to blot HA-tagged CHS3 co-expressed with V5-tagged CSA1 or HA-tagged CSA1 coexpressed with V5-tagged CHS3 to demonstrate the formation of oligomers of similar sizes (Fig. 1, C and D). These results suggest that sensor CHS3 and executor CSA1 from both clade 2 Per-0 and clade 3 Ws-2 formed the same size oligomers (Fig. 1, C and D). Overall, the sensor CHS3 and executor CSA1 from clade 2 or clade 3 oligomerize in a manner dependent on their respective pair partner and form the same size oligomers.

## CHS3-CSA1 TNL pairs form a hetero-oligomeric complex

**Fig. 2.**
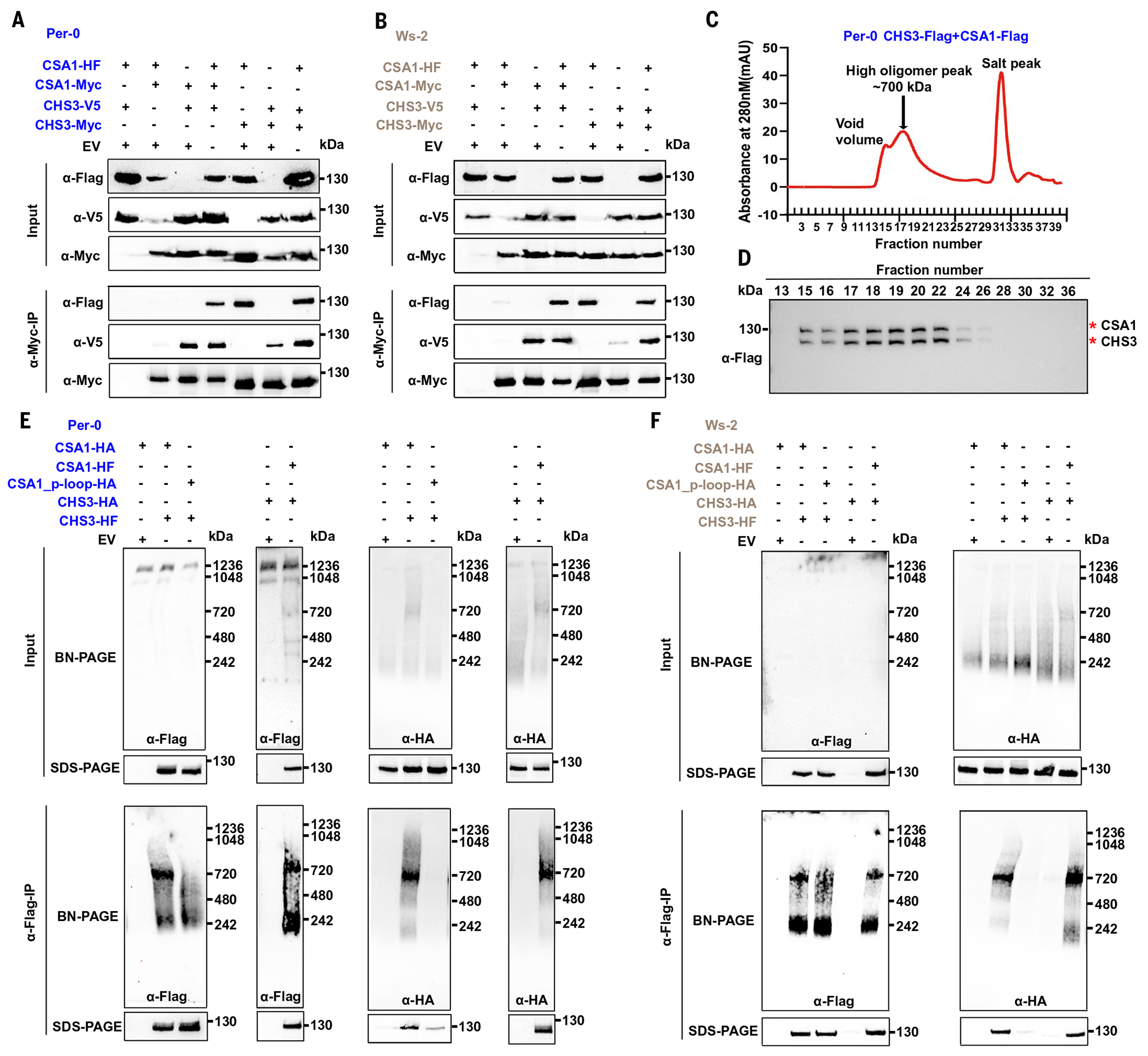
The CHS3-CSA1 TNL pair forms a hetero-oligomeric complex. (**A** and **B**) Self-association of CSA1 or CHS3 is weak in the absence of its partner in both clade 2 (A) and clade 3 (B). HF, 6×His-3×Flag. (**C**) SEC shows the high oligomer formation of the clade 2 Per-0 CHS3-CSA1 pair. SEC analysis of the purified CHS3-CSA1 pair proteins from clade 2 Per-0 on a Superose 6 Increase 10/300 GL column. (**D**) Western blot demonstrating the coelution of sensor CHS3 and executor CSA1 from clade 2 Per-0. Western blot analysis of the distribution of CHS3 and CSA1 from the SEC fractions in (C). (**E** and **F**) The CHS3-CSA1 pair forms a hetero-oligomeric complex. (E) Co-IP–BN-PAGE results for the clade 2 Per-0 CHS3-CSA1 pair. (F) Results for the clade 3 Ws-2 pair.

The above results suggest, but do not prove, that CHS3 and CSA1 form a hetero-oligomer. If true, then the proposed oligomeric state of the CHS3-CSA1 TNL pair would be different from the homo-oligomerization of activated NRC class helper NLRs after sensor NLR activation (30–32). We performed coimmunoprecipitation (co-IP) assays to test for self-association of CHS3 and CSA1 in the presence or absence of the respective pair partner. We made HF- and Myc-tagged CHS3 or CSA1, and these new constructs functioned as previously reported (43) (fig. S2). We coexpressed two differently tagged CSA1s or CHS3s with either empty vector (EV) or with its partner and performed co-IP assays. We found that self-association of either CSA1 or CHS3 was weak or not detectable in the absence of its partner (Fig. 2, A and B, and fig. S3, A and B). Coexpression with the partner greatly increased the co-IP of differentially tagged CSA1 or CHS3 from either the clade 2 or clade 3 pair (Fig. 2, A and B, and fig. S3, A and B). Meanwhile, a heterocomplex containing both CHS3 and CSA1 was readily detected if they were coexpressed (Fig. 2, A and B). We then coexpressed the CHS3-CSA1 pair from clade 2 Per-0 in insect cells and purified the proteins for size-exclusion chromatography (SEC) (fig. S3, C and D). We found that purified clade 2 Per-0 CSA1 and CHS3 proteins formed a high–molecular weight complex that coeluted in fractions from a size-exclusion column consistent with hetero-oligomer formation (Fig. 2, C and D). We performed co-IP followed by BN-PAGE to further determine whether sensor CHS3 and executor CSA1 form separate homo-oligomers or a hetero-oligomeric complex. When we immunopre-cipitated either HF-tagged sensor CHS3 or executor CSA1, we detected the oligomer consistently, whether we were blotting for the sensor or its executor partner in both clade 2 Per-0 and clade 3 Ws-2 (Fig. 2, E and F). The P-loop mutant of CSA1, which was extremely weakly coimmunoprecipitated with CHS3, was used as a negative control. These data indicate that CHS3 and CSA1 oligomerize and form a hetero-oligomeric complex—and not separate homo-oligomers—to function.

## Conserved and clade-specific TIR domain features are required for CHS3-CSA1 function

TNLs, such as RPP1 and ROQ1, oligomerize and form a tetramer upon activation (21, 22). The structures of RPP1 and ROQ1 showed that the TIR domains engage in a head-to-head symmetric interaction involving the alpha helices αA and αE, called the AE interface, after which further head-to-tail asymmetric interaction is induced, reorganizing the asymmetric BB-loop (21, 22). These two interactions in TIR domains are essential to align the TIR domains in a conformation conducive to NADase active-site function (21, 22). Several previous studies have defined requirements of the AE interface and BB-loop in TIR domain function (45–49). On the basis of the co-IP and BN-PAGE results (Fig. 2), we hypothesized that the majority of TNL CHS3 and CSA1 proteins first associate to form heterodimers, which then dimerize to a heterotetrameric oligomer. If our hypothesis is correct, then the AE interface in the TIR domains of both sensor CHS3 and executor CSA1 should be essential to mediate head-to-head interaction, but only the BB-loop in the TIR domain of executor CSA1, which contains conserved catalytic residue, should be required for head-to-tail interaction (fig. S4A).

**Fig. 3.**
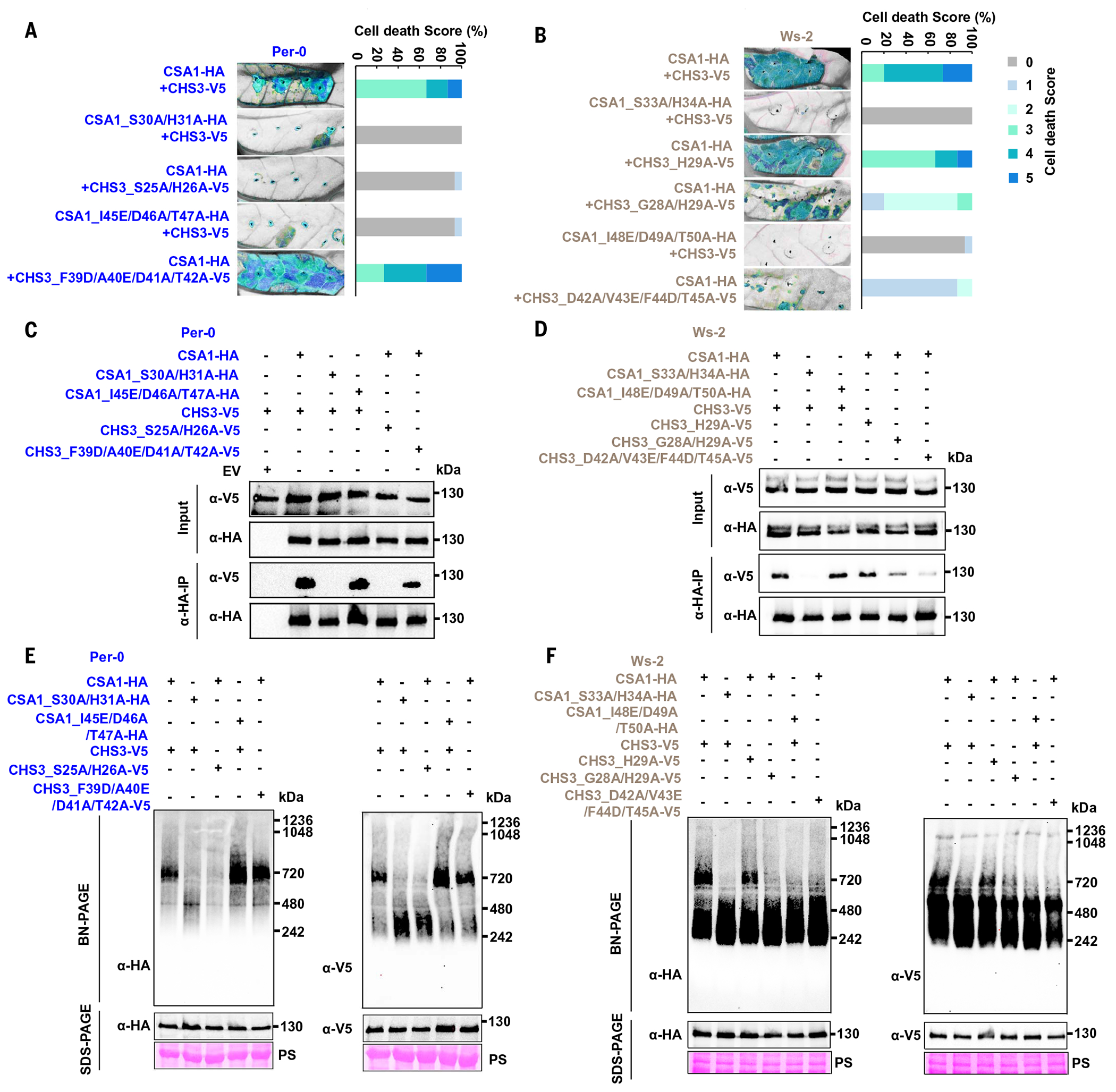
CHS3-CSA1 TNL pair function and oligomerization require both conserved and clade-specific TIR domain features. (**A** and **B**) In planta (*N. tabacum*) cell death phenotypes (left) and the corresponding percentage representation of cell death scores (right). Cell death scoring is described in fig. S5A. Images were photographed under UV light at 4 to 5 dpi. Dead tissue is shown in cyan, and live tissue is shown in gray (here and in subsequent figures). Conserved residues in the TIR domain AE interface in both CSA1 and CHS3 and the function of the CSA1^TIR^ BB-loop are required for cell death induction in clade 2 (A) and clade 3 (B). The CHS3^TIR^ BB-loop of clade 2 Per-0 is dispensable for cell death induction (A), but the CHS3^TIR^ BB-loop of clade 3 Ws-2 is required for cell death induction (B). Stacked bars are color-coded showing the proportions (in percentage) of each cell death score (0 to 5). Fifteen leaves were scored for each stacked bar. Clade 2 accession and proteins are in blue, and clade 3 accession and proteins are in gray. (**C** and **D**) Co-IP assays show the effects of mutations in the AE interfaces and BB-loops of CHS3^TIR^ and CSA1^TIR^ on protein interaction. (C) Conserved residues in the TIR domain AE interfaces of CSA1 and CHS3 are required for interaction of the clade 2 Per-0 pair, but residues in the BB-loop are dispensable. (D) By contrast, although conserved residues in the AE interface are also required for interaction of the Ws-2 clade 3 pair, the CHS3^TIR^ BB-loop is also essential. (**E** and **F**) BN-PAGE assays show the effects of TIR domain AE interface and BB-loop mutations of CSA1 and CHS3 on oligomerization. (E) Conserved residues in the TIR domain AE interfaces of CSA1 and CHS3 are required for oligomerization of the clade 2 Per-0 pair, but residues in the BB-loops are dispensable. (F) By contrast, residues in both the AE interfaces and the BB-loops of CSA1^TIR^ and CHS3^TIR^ are required for oligomerization of the clade 3 Ws-2 pair.

We aligned the full-length protein sequences of CHS3 and CSA1 from different clades with other plant TNLs to identify conserved residues within the AE interface and BB-loop. We then introduced mutations in the AE interfaces and BB-loops of CHS3^TIR^ and CSA1^TIR^ (fig. S4, B and C). We made these mutations based on either previous studies reported or the charge and side chain of amino acid (50, 51) (table S1). Consistent with published studies on plant TNLs, mutating two conserved residues (SH to AA) in the αA helix of CSA1^TIR^ from clade 2 Per-0 or clade 3 Ws-2 completely abolished CHS3-CSA1 pair-mediated cell death (Fig. 3, A and B; cell death scale is shown in fig. S5A). The protein sequence alignments showed that the sensor CHS3 from clade 2 Per-0 contains two conserved residues (SH) in the αA helix of the TIR domain, whereas clade 3 Ws-2 CHS3 only contains one conserved residue (H) (fig. S4B). Mutating SH to AA in clade 2 Per-0 CHS3^TIR^ abrogated cell death (Fig. 3A). By contrast, an H-to-A single mutation in clade 3 Ws-2 CHS3^TIR^ only slightly decreased cell death (Fig. 3B). We then generated a GH-to-AA double mutation in Ws-2 CHS3^TIR^ and found that the cell death phenotype triggered by the clade 3 Ws-2 CHS3-CSA1 pair was largely suppressed (Fig. 3B). Thus, as expected, the AE interfaces of both CSA1^TIR^ and CHS3^TIR^ are required for function.

We then investigated the functional roles of the BB-loops of CSA1^TIR^ and CHS3^TIR^. The residues of the CSA1^TIR^ BB-loop are conserved across all clades, but those of the CHS3^TIR^ BB-loop are not (fig. S4C). Therefore, we mutated several conserved residues in the CSA1^TIR^ BB-loops and some residues at equivalent positions in CHS3^TIR^ BB-loops (table S1). We found that an IDT-to-EAA mutation in the CSA1^TIR^ BB-loop abolished cell death elicited by the clade 2 Per-0 or clade 3 Ws-2 CHS3-CSA1 pair (Fig. 3, A and B). In addition, we generated a G-to-A mutation in the CSA1^TIR^ BB-loop, a highly conserved BB-loop residue that is essential for ROQ1-mediated cell death (22) (fig. S4C). This mutation also resulted in the loss of CHS3-CSA1 pair–mediated cell death in both clade 2 and clade 3 pairs (fig. S5, B and C). These results are consistent with the formation of a CHS3-CSA1 TNL pair resembling the RPP1- or ROQ1-like heterotetramer, where the CSA1^TIR^ BB-loop is required for NADase active-site opening. We mutated four residues (FADT-to-DEAA) in the clade 2 Per-0 CHS3^TIR^ BB-loop and coexpressed this CHS3 mutant with Per-0 CSA1 to assess cell death phenotype. We found that this BB-loop mutant of clade 2 Per-0 CHS3 with CSA1 retained strong cell death induction (Fig. 3A). However, when we introduced a four-residue (DVFT-to-AEDA) mutation into the clade 3 Ws-2 CHS3^TIR^ BB-loop and coexpressed it with Ws-2 CSA1, the cell death phenotype was strongly decreased (Fig. 3B). These data suggest that, despite the close relatedness of these two CHS3-CSA1 TNL pairs, the BB-loop of clade 2 Per-0 CHS3 is dispensable for cell death induction, whereas that of clade 3 Ws-2 CHS3 is essential.

Next, we performed co-IP and BN-PAGE experiments comparing wild-type CHS3 and CSA1 with these mutant alleles from both clade 2 and clade 3 CHS3-CSA1 pairs to further explore TIR domain structural requirements for heterodimer formation and oligomerization. The SH-to-AA mutation in the TIR domain AE interface of either clade 2 Per-0 CSA1 or CHS3 led to loss of CHS3-CSA1 interaction and oligomerization (Fig. 3, C and E), consistent with this mutant’s loss of cell death phenotype (Fig. 3A). Similarly, mutation in the AE interface of clade 3 Ws-2 CSA1 resulted in loss of protein interaction and oligomerization (Fig. 3, D and F, and fig. S6), consistent with a requirement of these residues for function and parallel to the corresponding clade 2 Per-0 mutant. The single mutation in the AE interface of clade 3 Ws-2 CHS3 had no obvious effect on heterodimer formation, but the double mutation strongly decreased heterodimer formation (Fig. 3D). The H-to-A single mutation in the clade 3 Ws-2 CHS3^TIR^ AE interface slightly decreased CHS3-CSA1 oligomerization (Fig. 3F and fig. S6), whereas GH-to-AA double mutation almost abolished oligomerization (Fig. 3F). Thus, the TIR domain AE interfaces of both clade 2 and clade 3 CSA1-CHS3 pair are uniformly required for function, heterodimer formation, and oligomerization.

We further investigated the effects of BB-loops on heterodimer formation and oligomerization. We found that the mutants of the clade 2 Per-0 CSA1^TIR^ BB-loop and the CHS3^TIR^ BB-loop retained CHS3-CSA1 heterodimer formation and oligomerization (Fig. 3, C and E), despite our observation that the CSA1^TIR^ BB-loop mutant failed to induce cell death (Fig. 3A). Thus, these TIR domain features are required for clade 2 CSA1 function but are dispensable for heterodimer formation and oligomerization. The IDT-to-EAA mutation in the clade 3 Ws-2 CSA1^TIR^ BB-loop retained heterodimer formation (Fig. 3D) but lost oligomerization (Fig. 3F and fig. S6), consistent with its full loss of cell death phenotype. Thus, the CSA1 clade 3 BB-loop is uniquely required for both function and oligomerization. The G-to-A mutation in both the clade 2 Per-0 and clade 3 Ws-2 CSA1^TIR^ BB-loops retained oligomerization (fig. S5, D and E). Mutation of the clade 3 Ws-2 CHS3^TIR^ BB-loop nearly abolished heterodimer formation and oligomerization (Fig. 3, D and F, and fig. S6), consistent with this mutant’s effect on cell death induction (Fig. 3B). Thus, the requirement for the CHS3^TIR^ BB-loop in function, protein interaction, and oligomerization in clade 3 Ws-2 contrasts with clade 2 Per-0, which does not require the CHS3^TIR^ BB-loop.

**Fig. 4.**
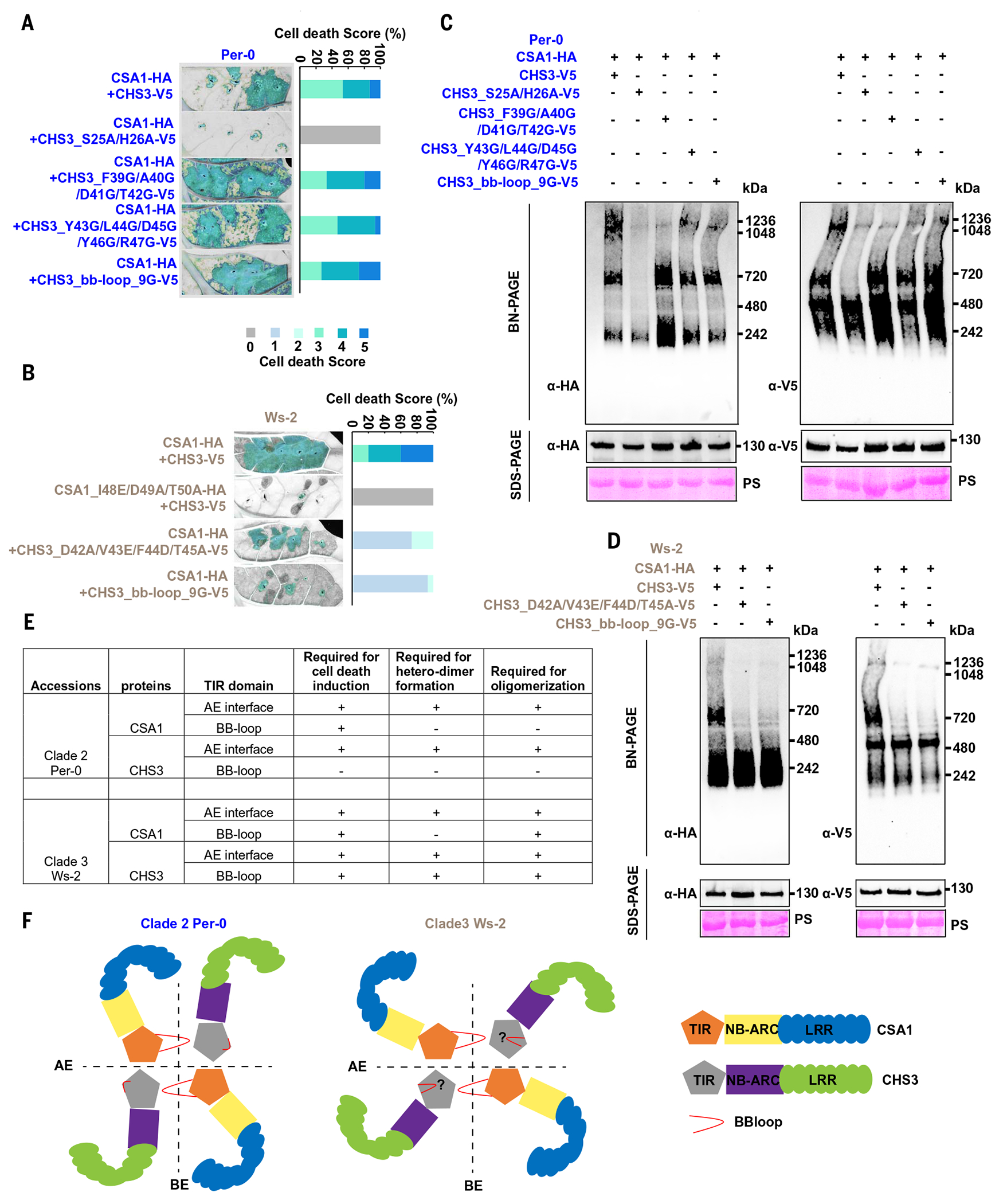
Differential requirements for function and oligomerization between the CHS3^TIR^ BB-loops from clade 2 and clade 3. (**A** and **B**) In planta (**N. tabacum**) phenotypes (left) and corresponding percentage representations of cell death score (right). The mutations in the CHS3^TIR^ BB-loop of clade 2 Per-0 cannot influence clade 2 CHS3-CSA1 pair–triggered cell death (A); by contrast, the mutations in the CHS3^TIR^ BB-loop of clade 3 Ws-2 largely decreased cell death induction (B). Images were photographed at 4 to 5 dpi. Clade 2 accession and proteins are in blue, and those of clade 3 are in gray. Stacked bars are color-coded showing the proportions (in percentage) of each cell death score (0 to 5). Fifteen leaves were scored for each stacked bar. (**C** and **D**) The CHS3^TIR^ BB-loop is dispensable for oligomerization of clade 2 Per-0 pair (C) but indispensable for clade 3 Ws-2 pair oligomerization (D). (**E**) Summary of CHS3-CSA1 TNL pair requires both conserved and clade-specific TIR domain features. A plus sign indicates that this TIR domain feature is required, and a minus sign indicates that it is not required for each function listed. Note especially the differences in clade-specific requirements for the CHS3 BB-loops. (**F**) Schematic representation of the predicted structures of CHS3-CSA1 pairs from clade 2 Per-0 and clade 3 Ws-2. The AE interfaces of CSA1 and CHS3 are required for head-to-head interaction of CSA1^TIR^ and CHS3^TIR^ and promote heterodimer formation. The CSA1^TIR^ BB-loop mediates head-to-tail interaction of CSA1 and CHS3 TIR domains to promote the dimerization of heterodimers. Based on the analysis of mutations detailed above, there should be differences in the structures of clade 2 Per-0 and clade 3 Ws-2. LRR, leucine-rich repeat.

To confirm differential requirements for function and oligomerization between the CHS3^TIR^ BB-loops from clade 2 and clade 3, we made more mutations in CHS3^TIR^ BB-loops to assess cell death phenotype and oligomerization of clade 2 and clade 3 pairs (table S1). We generated four-residue (FADT-to-GGGG), five-residue (YLDYR-to-GGGG), and nine-residue (FAD-TYLDYR-to-GGGGGGGGG) mutations in the clade 2 Per-0 CHS3^TIR^ BB-loop and then co-expressed these CHS3 mutants with Per-0 CSA1. Meanwhile, we mutated nine residues (FTNGISRDQ-to-GGGGGGGGG) in the clade 3 Ws-2 CHS3^TIR^ BB-loop at the equivalent position to the clade 2 Per-0 CHS3^TIR^ BB-loop and coexpressed it with Ws-2 CSA1. Consistent with results noted above, the CHS3^TIR^ BB-loop is required for cell death induction and oligomerization of the clade 3 Ws-2 pair but not that of the clade 2 Per-0 pair (Fig. 4, A to D).

We also transiently expressed wild-type or mutant CHS3-CSA1 pairs from either clade 2 Per-0 or clade 3 Ws-2 in *Arabidopsis* leaves to test cell death induction and oligomerization. Our results regarding structural requirements for function and oligomerization confirmed those established in *Nicotiana benthamiana* (fig. S7). Altogether, our mutant analyses suggest that the AE interfaces of CSA1^TIR^ and CHS3^TIR^ are required for head-to-head interaction of CSA1 and CHS3 TIR domains to promote heterodimer formation. We propose that the BB-loop of CSA1 mediates head-to-tail interaction of TIR domains and promotes formation of a CHS3-CSA1 oligomer that is likely a dimer of heterodimers (Fig. 4, E and F). However, similar mutations in clade 2 Per-0 and clade 3 Ws-2 CSA1^TIR^ BB-loops had distinct effects on oligomerization (Fig. 4E). The clade 2 Per-0 CHS3^TIR^ BB-loop is dispensable for cell death induction, heterodimer formation, and oligomerization, but the clade 3 Ws-2 CHS3^TIR^ BB-loop is essential for all three processes (Fig. 4E). Thus, the phenotypic consequences of mutations in the clade 2 Per-0 CHS3-CSA1 TNL pair follow expectations based on the RPP1- or ROQ1-like tetrameric structures, but this is not true for the clade 3 Ws-2 pair. Therefore, our results are consistent with the hypothesis that the AE interface in the TIR domain of CHS3 and CSA1 mediates the head-to-head interaction, and the BB-loop of the TIR domain mediates the head-to-tail interaction, eventually forming a novel heterotetrameric oligomer, but the oligomerization and structure of even closely related CHS3-CSA1 paired TNLs are likely diverse (Fig. 4F).

## BAK1 and/or BIRs suppress the oligomerization of CHS3-CSA1 TNL pairs

**Fig. 5.**
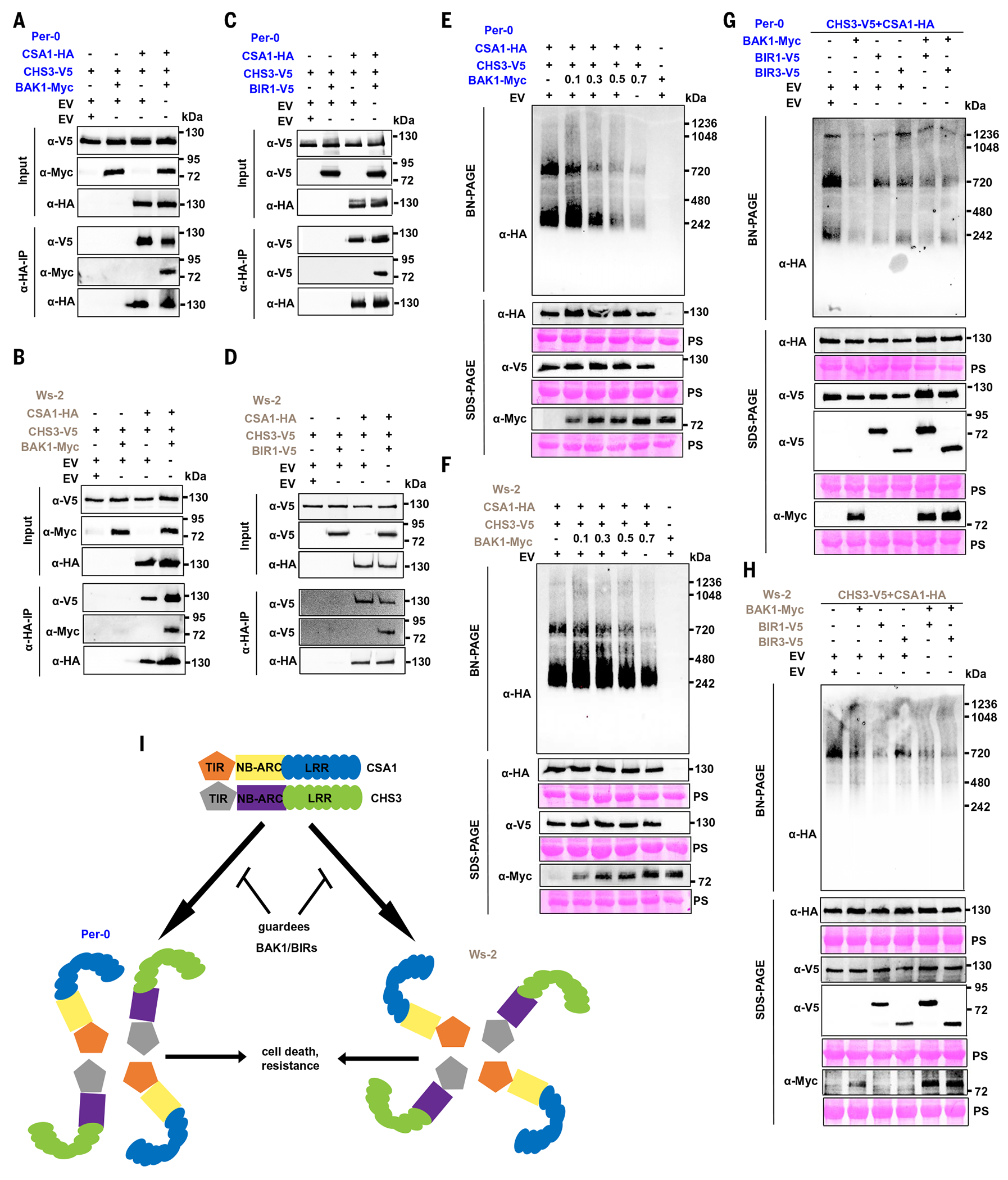
BAK1 and/or BIRs suppress the oligomerization of the CHS3-CSA1 TNL pair but not heterodimer formation. (**A** to **D**) Co-IP assays showing that coexpression of BAK1 [(A) and (B)] or BIR1 [(C) and (D)] does not affect the interaction of CSA1 and CHS3 from either clade 2 Per-0 [(A) and (C)] or clade 3 Ws-2 [(B) and (D)]. (**E** and **F**) BAK1 coexpression inhibits oligomerization of the CHS3-CSA1 pair from clade 2 Per-0 (E) or clade 3 Ws-2 (F). The suppression of oligomerization correlates with increased expression of BAK1 (numbers represent OD = 600 nm). (**G** and **H**) BIR1 or BIR3 coexpression moderately inhibits oligomerization of the CHS3-CSA1 pair from clade 2 Per-0 (G) or clade 3 Ws-2 (H). (**I**) Schematic of the regulation of CHS3-CSA1 TNL pair oligomerization by the BAK1 or BIR proteins. In the resting state, the clade 2 or clade 3 CHS3-CSA1 TNL pair forms heterodimers and cannot further oligomerize because of negative regulation by the BAK1 and/or BIRs. Upon modulation of the BAK1 and/or BIRs by effectors or other mechanisms, suppression is relieved, and we speculate that the CHS3-CSA1 pair forms a dimer of heterodimers to activate immune response and cell death.

BAK1 acting as an immune co-receptor plays a critical role in pattern-triggered immunity (PTI) signaling and is targeted by a type III effector, HopB1 (52). Recent studies have demonstrated that the clade 1 Col-0 CSA1 is required for the autoimmune phenotype of *bak1 bkk1* and *bak1 bir3* (43, 53) and that BAK1, BIR1, and BIR3 also inhibit the cell death phenotype induced by the CHS3-CSA1 pair from clade 2 or clade 3 (43). These data indicate that BAK1 and BIR proteins maintain the CHS3-CSA1 pairs in an inactive state across all clades, presumably acting as guardees until effector activation. The precise mechanism of this negative regulation is unknown. We investigated whether BAK1 and/or BIRs could regulate the heterodimer formation or oligomerization of clade 2 and clade 3 CHS3-CSA1 pairs. We first coexpressed each CHS3-CSA1 pair with either the EV or BAK1 and/or BIRs and performed co-IPs. We found that BAK1 and/or BIRs did not affect the interaction between CHS3 and CSA1 for either the clade 2 or clade 3 pair (Fig. 5, A to D, and fig. S8). We then used BN-PAGE assays to test whether BAK1 or BIR proteins modulate CHS3-CSA1 pair oligomerization. Coexpression of either CHS3-CSA1 pair with BAK1 suppressed the oligomerization of either CHS3-CSA1 pair in both *N. benthamiana* and *Arabidopsis* (Fig. 5, E and F, and fig. S9), and the suppression was correlated with BAK1 concentration (Fig. 5, E and F). Similarly, BIRs also inhibited the oligomerization of CHS3-CSA1 pairs in a dose-dependent manner (Fig. 5, G and H, and fig. S10). In addition, consistent with our previous findings (43), coexpression of BAK1 and BIRs with a CHS3-CSA1 pair showed greater inhibition of oligomer formation compared with BIRs alone (Fig. 5, G and H).

BAK1 and BIR1 are RLKs, and they have kinase activity (54–57). To examine whether the kinase activity of BAK1 or BIR1 is required for the inhibition of CHS3-CSA1 pair oligomerization, we generated mutants of BAK1 and BIR1 that are known to abolish or reduce their kinase activities (54–57) and coexpressed them with the CHS3-CSA1 pair from either clade 2 Per-0 or clade 3 Ws-2. All kinase-inactive mutants of BAK1 retained the ability to suppress CHS3-CSA1 pair–mediated cell death and oligomerization (fig. S11). Additionally, the BIR1 K331E mutant, which markedly reduced the kinase activity of BIR1 (57), also retained inhibition of cell death induction and oligomerization of the CHS3-CSA1 pairs (fig. S12). Taken together, BAK1 and/or BIRs, likely acting as guardees, inhibit CHS3-CSA1 oligomerization but not the initial CHS3-CSA1 heterodimerization. We propose that this maintains the preexisting heterodimer in an inactive state in the absence of a pathogen (Fig. 5I). Notably, the kinase activity of BAK1 or BIR1 is dispensable for this inhibition, which indicates that the CHS3-CSA1 pair has evolved to detect the presence or absence of its guardees rather than their activity.

## Discussion

Oligomerization of NLRs plays an essential role in defense activation and cell death induction (18–31). To date, the oligomeric state and structural requirements for the formation of activated oligomers have not been addressed for paired NLRs, a major subtype of plant intracellular innate immune receptors. The TNL pair RRS1-RPS4 (RESISTANCE TO RALSTONIA SOLANACEARUM1-RESISTANCE TO P.SYRINGAE 4) (evolutionarily related to the CHS3-CSA1 pair) was thought to be unable to form a RPP1- or ROQ1-like heterotetramer because the sensor RRS1 does not contain a conserved catalytic Glu residue in its TIR domain, and its BB-loop was thought to be too short to support RPP1^TIR^-like asymmetric homodimer formation (21). However, we found that activated CHS3-CSA1 TNL pairs from two closely related clades oligomerize and form a hetero-oligomeric complex (<720 kDa) that we speculate is a dimer of heterodimers tetrameric complex. This predicted structure of the TNL CHS3-CSA1 pair contains two NADase active sites from the CSA1 executor, whereas the BB-loop of the CHS3 sensor is allowed to be disordered. Our speculation of a dimer of heterodimers is based on the predicted size of the oligomer (<720 kDa), TIR domain functional requirements, and the precedent of the RPP1 and ROQ1 structures.

We demonstrate that clade 2 and clade 3 pairs form distinguishable hetero-oligomeric complexes through analysis of the requirements for their respective TIR domain AE interfaces and BB-loops in the oligomerization and function. The residues in the AE interfaces of CHS3^TIR^ and CSA1^TIR^ and the residues in the CSA1^TIR^ BB-loop are highly conserved across all clades. By contrast, the CHS3^TIR^ BB-loop features intraclade but not interclade conservation (fig. S13). We found that the requirements of the AE interfaces in clade 2 and clade 3 proteins are similar but that there are distinct requirements for the CSA1^TIR^ and CHS3^TIR^ BB-loops between clade 2 and clade 3.

It is noteworthy that the conserved residues (SH) in the AE interfaces of the sensor RRS1^TIR^ and the executor RPS4^TIR^ are required for effector-mediated cell death but are not required for the interaction between full-length RRS1 and RPS4 proteins (45). This is in contrast to the requirements for the AE interfaces in the TIR domains of the CHS3-CSA1 pair, which are required for all processes (cell death induction, heterodimer formation, and oligomerization). Taken together, these data suggest that there are diverse oligomerization requirements and consequently diverse structural configurations in NLR pairs and that overgeneralizations from single paired NLR structures may fail to capture existing structural heterogeneity.

We also demonstrate that BAK1 and BIRs inhibit the function of both clade 2 and clade 3 CHS3-CSA1 TNL pairs before oligomerization but after heterodimer formation. We found that the kinase activity of BAK1 and BIR1 is dispensable for negative regulation of CHS3-CSA1 function and oligomerization, and previous studies have demonstrated that the kinase activity of BAK1 is required for depletion of BAK1 by the type III virulence effector HopB1 (52). Therefore, BAK1 kinase activity is dispensable for inhibition of CHS3-CSA1 pair cell death function and oligomerization; however, the kinase activity of BAK1 is ultimately essential for effector-dependent CHS3-CSA1 pair activation through BAK1 depletion, which we suggest then allows oligomerization to proceed to full CHS3-CSA1 activation. Our data provide a template to interpret differential functional consequences of structural heterogeneity across paired NLRs.

## Materials and methods

### Plant material and growth condition

*N. benthamiana* and *N. tabacum* were grown in a growth chamber with 24°C/20°C and 16 hours/8 hours light/dark cycle on mixed soil. *Arabidopsis thaliana* Col-0 were grown in a climate-controlled growth room at 22°C/18°C with 8 hours/16 hours light/dark photoperiod on a mix of potting soil and sand.

### Plasmid constructions

The constructs of C-terminally HA-tagged clade 2/3 CSA1, V5-tagged clade 2/3 CHS3, Myc/V5-tagged BAK1, and V5-tagged BIRs used in this study are same as those used in our previous study (43). For new CSA1-CHS3 constructs either fused with new tags or carrying specific mutations, the genomic fragments of CSA1 and CHS3 were polymerase chain reaction (PCR) amplified from previous recombinant vectors, then the new constructs were generated using the Golden Gate assembly cloning procedure described previously (39, 43). Briefly, the full-length genomic sequence was directly amplified or based on the positions of the amino acids we mutated, the full-length genomic sequence was split into two fragments for amplification, and some tags, such as HF (6×His-3×Flag) or Myc, were amplified using other vectors as templates. All PCR products were cloned into the binary vector pICSL86922 with 35S promoter and the TMV omega translational enhancer. Site-directed mutants were generated by PCR mutagenesis as described previously (43). All PCR primers and the resulting products are defined in table S2.

### *Agrobacterium*-mediated transient expressions

The proteins of interest were transiently expressed in *N. benthamiana* according to a previously described method (43). *Agrobacterium tumefaciens* strain GV3101 carrying the indicated constructs were grown for overnight in a 28°C shaking incubator, the bacteria were collected by centrifugation and resuspended in infiltration buffer [10 mM MES (pH 5.6), 10 mM MgCl_2_, and 100 μM acetosyringone]. Unless otherwise noted in the text, the final infiltration OD_600_ (optical density at 600 nm) used was 0.5 for each strains carrying vectors to be expressed. Leaves of 4- to 5-week-old *N. benthamiana* and *N. tabacum* were infiltrated with a 1-ml needle-less syringe. Plants were put back into the growth chamber at 24°C/20°C and a 16 hours/8 hours light/dark cycle after inoculation, and cell death phenotypes were photographed at 4 to 5 days postinfiltration (dpi). Leaves were harvested for immunoblots, coimmunoprecipitation, and BN-PAGE experiments at 2 dpi.

Transient expression of the indicated proteins in *Arabidopsis* Col-0 leaves was based on a previously described method (58). Briefly, *A. tumefaciens* strain GV3101 carrying the indicated constructs were grown onto YEB plates for 2 days in a 28°C incubator. The *Agrobacteria* were harvested from YEB plates and transferred to washing solution (10 mM MgCl_2_ and 100 μM acetosyringone), then the OD_600_ was measured. The *Agrobacteria* were diluted to OD_600_ = 0.8 for each strain in infiltration buffer (¼ MS, 1% sucrose, 100 μM acetosyringone and 0.01% silwet). Leaves of 3- to 4-week-old *Arabidopsis* plants were infiltrated with a 1-ml needle-less syringe. The *Arabidopsis* plants were moved into dark for 24 hours, then put back into the growth chamber at 22°C/18°C with 8 hours/16 hours light/dark cycle. The cell death phenotypes were photographed at 6 to 7 dpi, and leaves were harvested for BN-PAGE experiments at 4 dpi.

### Protein extraction for BN-PAGE and corresponding SDS-PAGE

*A. tumefaciens* strain GV3101 carrying the desired constructs were infiltrated into at least four leaves from different *N. benthamiana* plants or at least eight leaves from different *Arabidopsis* plants as described above. Then, six leaf disks for each sample were harvested into a 2-ml Eppendorf tube with three 4-mm glass beads at 2 dpi and flash frozen in liquid nitrogen. Frozen samples were ground in liquid nitrogen, and we then added 200 μl of extraction buffer [50 mM Tris, 50 mM NaCl, 5 mM MgCl_2_,10% glycerol, 10 mM dithiothreitol (DTT), 1× Sigma plant protease inhibitor cocktail (Sigma-Aldrich) and 1% Digitonin (Invitrogen)], rested samples on ice, and vortexed until all samples were liquid. Centrifugation was performed at 14,000 rpm for 5 min at 4°C, and the soluble supernatants were transferred to new 1.5-ml tubes. Centrifugation was performed again at 14,000 rpm for 15 min at 4°C to remove leftover cell debris. The clear lysate was used for BN-PAGE and corresponding SDS-PAGE experiments.

### BN-PAGE

For BN-PAGE, an equal volume of soluble supernatant of each sample extracted as detailed above was transferred to a new tube and diluted following the manufacturer’s instructions by adding Native PAGE 5% G-250 sample, 4× Sample Buffer (Invitrogen) and water. After this, the samples were immediately loaded on Native PAGE 3–12% Bis-Tris gels alongside NativeMark unstained protein standard (Invitrogen) to predict the size of the detected protein species and run at 150 V in dark buffer for 50 min followed by 250 V in light buffer for 1 hour. The proteins were then transferred to polyvinylidene difluoride (PVDF) membranes and were fixed to the membranes by incubating with 8% acetic acid for 15 min, washed with water three to four times, and put in a fume hood to dry for 20 min. Methanol was used to subsequently reactivate the membranes to correctly visualize the unstained native protein marker. After labeling the native protein marker on the membranes and washing the membranes with methanol and water several times, the membranes were immunoblotted as described below.

### SDS-PAGE and immunoblots

An equal volume of protein extract from each sample was transferred to a new tube and diluted with an equal volume of 5× SDS-PAGE loading buffer and denatured at 95°C for 8 min. Denatured samples were centrifuged at 12,000 rpm for 3 min and the supernatant was run on 8% SDS-PAGE gels. The proteins were then transferred to nitrocellulose (NC) membrane. The membranes were blocked with 5% milk dissolved in TBST (tris-buffered saline with Tween) for 1 hour at room temperature and subsequently incubated with desired antibodies at 4°C overnight. The following antibodies were used: anti-HA (Roche, cat. no. 11867431001, RRID: AB_390919), anti-Flag (Sigma, cat. no. F1804), V5-HRP (Sigma, V2260, RRID: AB_261857), Myc-HRP (Sigma, 16-213, RRID:AB_310809), HRP-conjugated anti-rat (Abcam, RRID: AB_10680316), and anti-mouse (Santa Cruz Biotechnology, cat. no. sc-516102). The enhanced chemiluminesence (ECL) substrate (cytiva) was used to detect the signals, and ponceau S solution was used to stain the membranes as loading control. The protein ladder (New England Biolabs) was used to show the molecular weight.

### co-IP

The co-IP experiments were performed as previously described (43). Leaves of *N. benthamiana* were harvested at 2 dpi and put into 2-ml tubes with four 4-mm glass beads, which were flash frozen in liquid nitrogen. Frozen samples were ground in liquid nitrogen and resuspended in 2 ml of extraction buffer (50 mM HEPES pH 7.5, 150 mM NaCl, 10 mM EDTA pH 8.0, 0.5% Triton X-100, 5 mM DTT with 1× plant protease inhibitor mixture) and mixed well using a vortex machine. Soluble supernatants were obtained by centrifugation twice at 10,400 *g* for 5 min and 20,800 *g* for 15 min at 4°C. Soluble supernatant of each sample was transferred to a new tube and mixed with 25 μl of anti-HA or anti-MYC conjugated magnetic beads (Miltenyi Biotec) and incubated for 2 hours with constant rotation at 4°C. The conjugated magnetic beads were captured using separation columns (Miltenyi Biotec) and were washed with washing buffer (50 mM HEPES pH 7.5, 150 mM NaCl, 10 mM EDTA pH 8.0, 0.2% Triton X-100, 5 mM DTT with 1× plant protease inhibitor mixture) three times. Proteins were eluted with 100 μl of elution buffer (Miltenyi Biotec). Proteins were resolved in 8% SDS-PAGE gels described above.

### Co-IP–BN-PAGE

A*grobacterium*-infiltrated leaves were collected into 2-ml tubes with four 4-mm glass beads at 2 dpi and flash frozen in liquid nitrogen. Frozen samples were then ground in liquid nitrogen. The 2 ml of extraction buffer [50 mM Tris, 50 mM NaCl, 5 mM MgCl2, 10% glycerol, 10 mM DTT, 1× Sigma plant protease inhibitor cocktail (Sigma-Aldrich) and 1% Digitonin (Invitrogen)] was added to each sample, then put on ice and vortexed until all samples were liquid. The samples were spun down at 14,000 rpm for 5 min at 4°C, and the lysate for each sample was transferred to new 2-ml tubes, then centrifugation was performed again at 14,000 rpm for 15 min at 4°C. The cleared lysate of each sample was mixed with 50 μl of Flag-M2 beads (Sigma) and inverted for 2 hours at 4°C. After incubation, centrifugation was performed at 2000 rpm for 2 min at 4°C and all supernatant was removed, then the beads were washed with protein extraction buffer three to four times. Elution was performed in 100 μl of extraction buffer with 0.4 mg/ml 3×Flag peptide (Sigma). Samples were incubated with constant rotation at 4°C for 1 hour shaking at 250 rpm once for 10 min after 30 min. Elution product of each sample was transferred to a fresh 1.5-ml tube, then, BN-PAGE and SDS-PAGE experiments were performed as detailed above.

### Ultraviolet (UV)–light imaging of cell death phenotypes and cell death scoring

The cell death phenotype and cell death scoring were measured based on our previously described methods (43). Briefly, 4 to 5 dpi leaves of *N. tabacum* were placed under UV lamps (B-100AP, UVP) and photographed using a digital camera (FUJIFLM, X-T1) with a yellow filter (B+W, 39, 022, 2x, MRC) in the camera lens. The cell death phenotype was scored according to the scale presented in our previous paper (43).

### Adjustment of red-green color combination in cell death phenotype

The leaves without cell death fluoresce red, and the leaves with cell death fluoresce green under UV light. The red-green color combination of the images were adjusted to aid figure visibility to the color vision deficient. The images in figures were grouped and merged as one image using Photoshop (Adobe, San Jose, CA) and under Hue/Saturation options: 90 points were increased to Red channel lightness; 130 points were increased to Yellow channel Hue; 60 points were increased to Green channel Hue; 100 points were reduced to Cyan channel Lightness. Eventually, the red-green color combination was changed to gray-cyan in cell death phenotype. The live tissue is shown in gray and dead tissue is shown in cyan.

### Purification of clade 2 Per-0 CSA1 and CHS3 for SEC analyses

Clade 2 Per-0 CSA1 was cloned into pFastBac-HTB vector with a C-terminal Flag tag, and Per-0 CHS3 was cloned into pFastBac1 vector with a C-terminal Flag tag. The two constructs were coexpressed in Sf9 insect cells for 72 hours. Cells were harvested and resuspended in buffer (50 mM Tris-HCl, pH7.5, 150 mM NaCl, 5 mM MgCl2, and 1 mM EDTA). After sonication and centrifugation at 20,000 *g* for 60 min, the CSA1 and CHS3 proteins were purified using Flag resin and eluted with 500 μg/ml 3×Flag peptide. Eluted proteins were concentrated and loaded onto a Superose 6 increase 10/300 GL column (GE healthcare) pre-equilibrated with buffer (10 mM Tris-Hcl, pH7.5, 150 mM NaCl, 1mM DTT). Peak fractions were subjected to Western blot analysis.

## Supplementary Material

MDAR statement

2
