# Supplementary material for: Paired plant immune CHS3-CSA1 receptor alleles form distinct hetero-oligomeric complexes
Source: Science. Author manuscript; Available in PMC 2024 Aug 5. (PMC11298796; doi:10.1126/science.adk3468)
Supplement: 2 [file NIHMS2010583-supplement-2.pdf]

Science
AAAS

Supplementary Materials for

Paired plant immune CHS3-CSA1 receptor alleles form distinct hetero-oligomeric complexes

Yu Yang *et al*.

Corresponding author: Jeffery L. Dangl, dangl@email.unc.edu

Science **383**, eadk3468 (2024)
DOI: 10.1126/science.adk3468

The PDF file includes:

Figs. S1 to S13
Tables S1 and S2

Other Supplementary Material for this manuscript includes the following:

MDAR Reproducibility Checklist

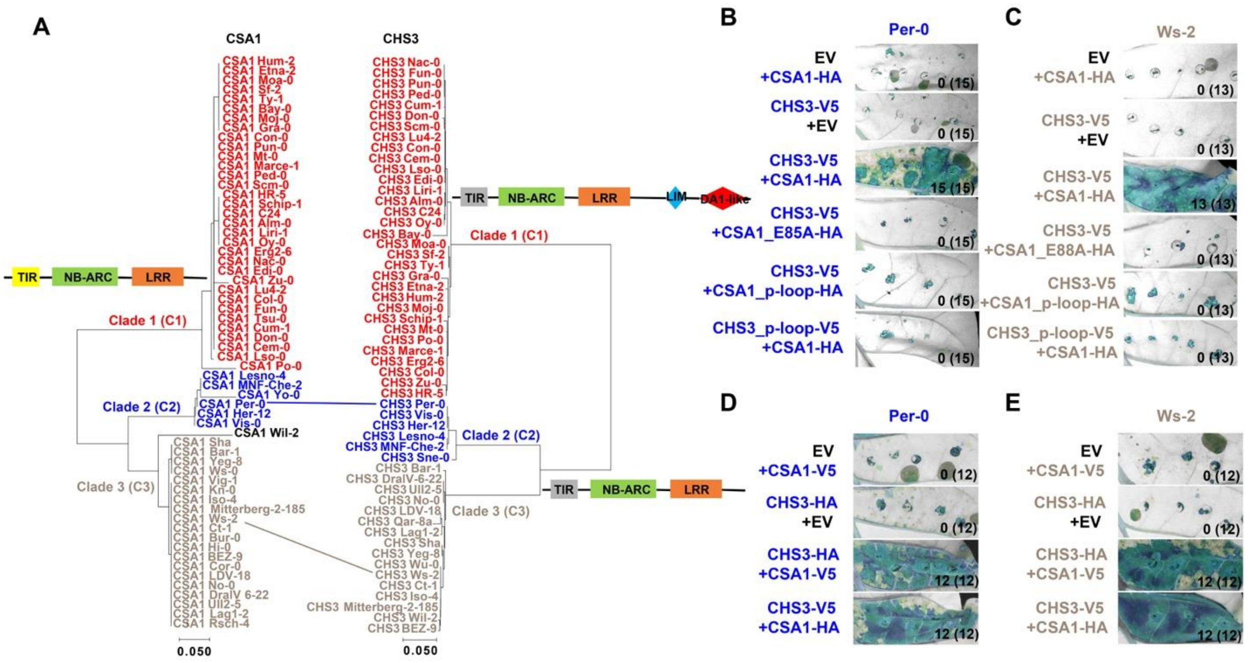

Fig. S1. Cell death phenotypes triggered by the CHS3-CSA1 TNL pairs in *N. tabacum*. (**A**) Phylogenetic tree of CSA1 (left) and CHS3 (right). Reused from Yang et al., (2022) for internal consistency. Clade 1 accessions are in red, clade 2 are in blue and clade 3 are in grey, respectively. Clade 2 accession Per-0 proteins and clade 3 accession Ws-2 proteins are used for this paper. (**B** and **C**) Co-expression of CHS3 and CSA1 from clade 2 Per-0 (B) or clade 3 Ws-2 (C) induces strong cell death, and the conserved catalytic Glu residue encoded in the CSA1 TIR domain and intact P-loops of both CSA1 and CHS3 are required. Dead tissue is shown in cyan and live tissue is shown in grey in this and all following figures. EV: empty vector. Clade 2 accession and proteins are in blue and clade 3 are in grey. The numbers indicate the numbers of leaves showing cell death out of the total number of leaves infiltrated. (**D** and **E**) Tag swapping does not affect CHS3-CSA1 TNL pair-mediated cell death in clade 2 Per-0 (D) and clade 3 Ws-2 (E). EV, empty vector. The numbers indicate the numbers of leaves displaying cell death out of the total number of leaves infiltrated.

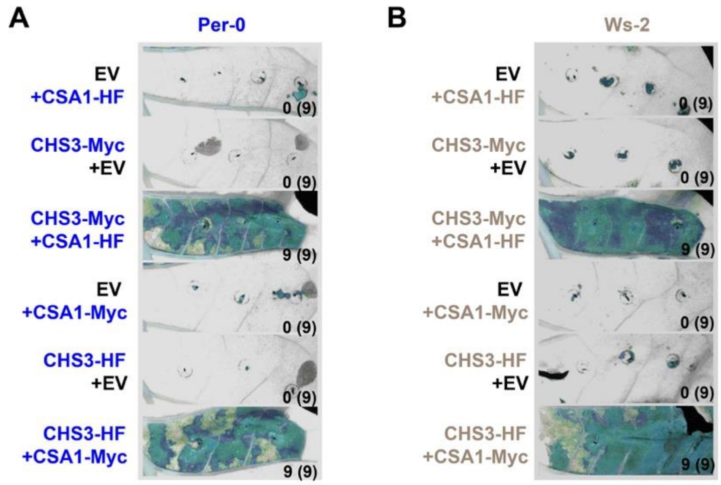

Fig. S2. Co-expression of CHS3 and CSA1 fused to different tags elicits similar cell death phenotype in *N. tabacum*. (**A** and **B**) The cell death phenotypes induced by new CHS3 and CSA1 constructs made in this work for self-association test and co-IP-BN-PAGE experiments. The clade 2 Per-0 is shown in (A) and clade 3 Ws-2 is shown in (B). EV, empty vector. Clade 2 accession and proteins are in blue and clade 3 are in grey. The numbers indicate the numbers of leaves displaying cell death out of the total number of leaves infiltrated.

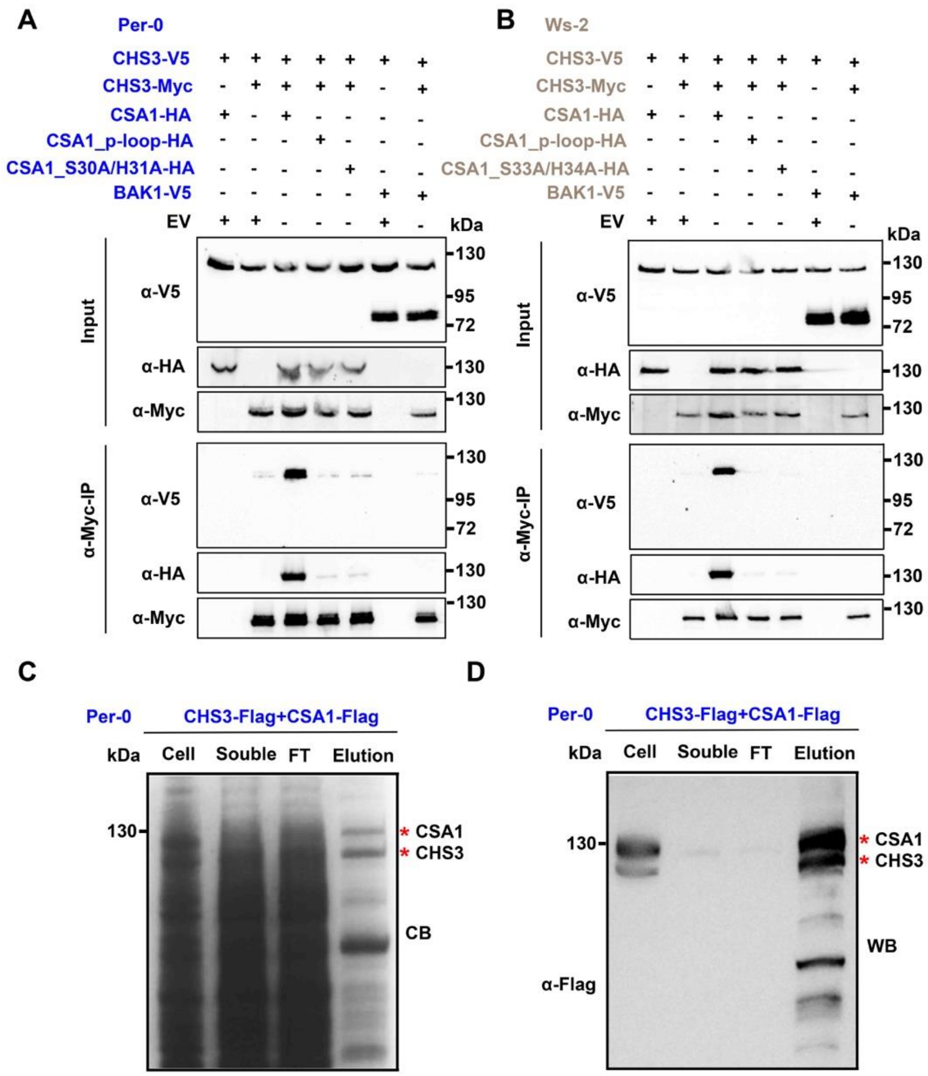

Fig. S3. Self-association of CHS3 and purification of the CHS3 and CSA1 proteins. (**A** and **B**) Self-association of CHS3 is very weak in the absence of its partner. Only wild-type CSA1 co-expression, but not loss-of-function mutants or BAK1 co-expression, is able to increase the co-IP of CHS3-V5 and CHS3-Myc from either clade 2 Per-0 (A) or clade 3 Ws-2 (B). (**C** and **D**) Coomassie blue staining (C) and western blot (D) show the protein purification. Flag-tagged CSA1 and CHS3 of clade 2 Per-0 were co-expressed in Sf9 insect cells and purified by Flag resin. CB, Coomassie blue. WB, western blot. FT, flow through.

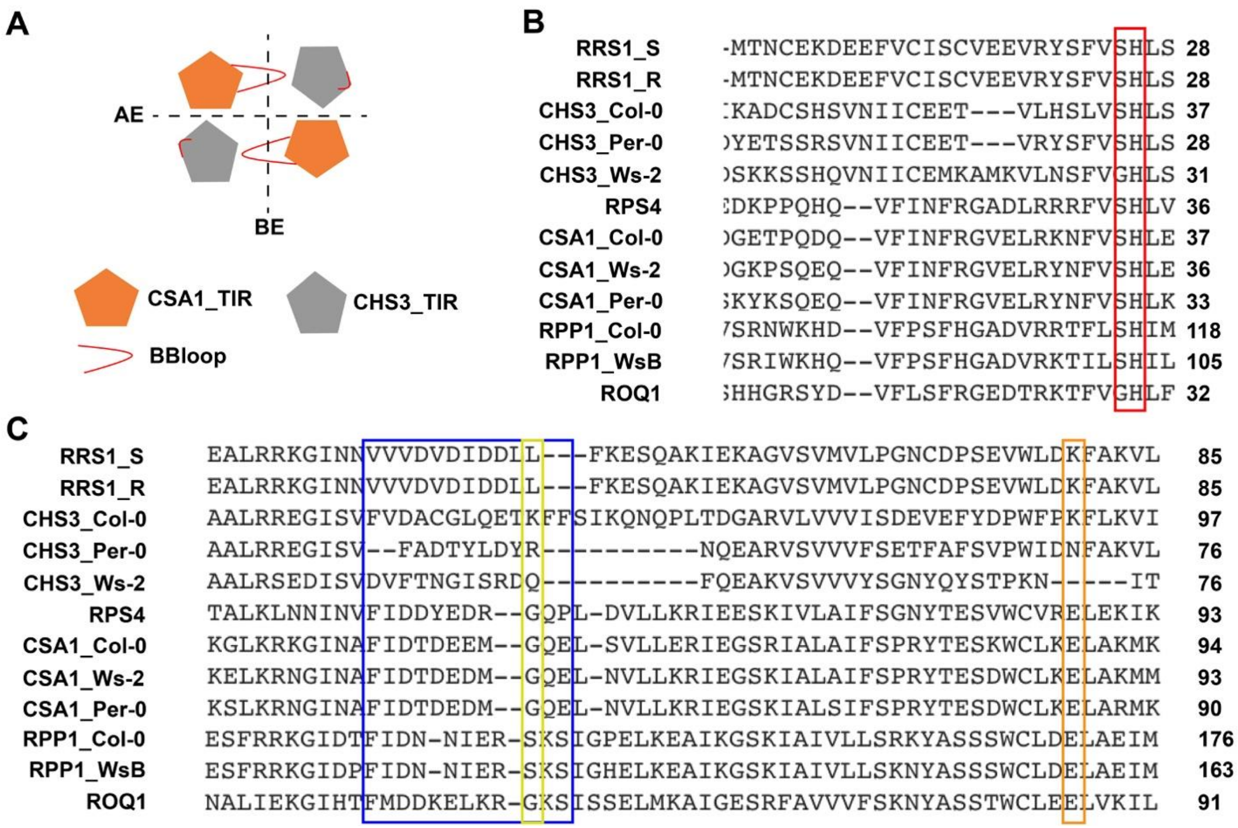

Fig. S4. Focused sequence alignments of various TNLs. (**A**) Schematic representation of the predicted structures of CHS3-CSA1 TIR domains. (**B** and **C**) Amino acid sequence alignment of the indicated TNLs showing the two conserved residues in TIR domain AE interface (B) and the equivalent positions of BB-loop and catalytic glutamic acid residue (C). The red box in (B) indicates the two conserved residues in AE interface, and the blue box in (C) indicates the position of BB-loop. The yellow box shows the residue at equivalent position with ROQ1 G52 in the BB-loop, which is required for the ROQ1-mediated cell death. The predicted catalytic glutamic acid is indicated by orange box.

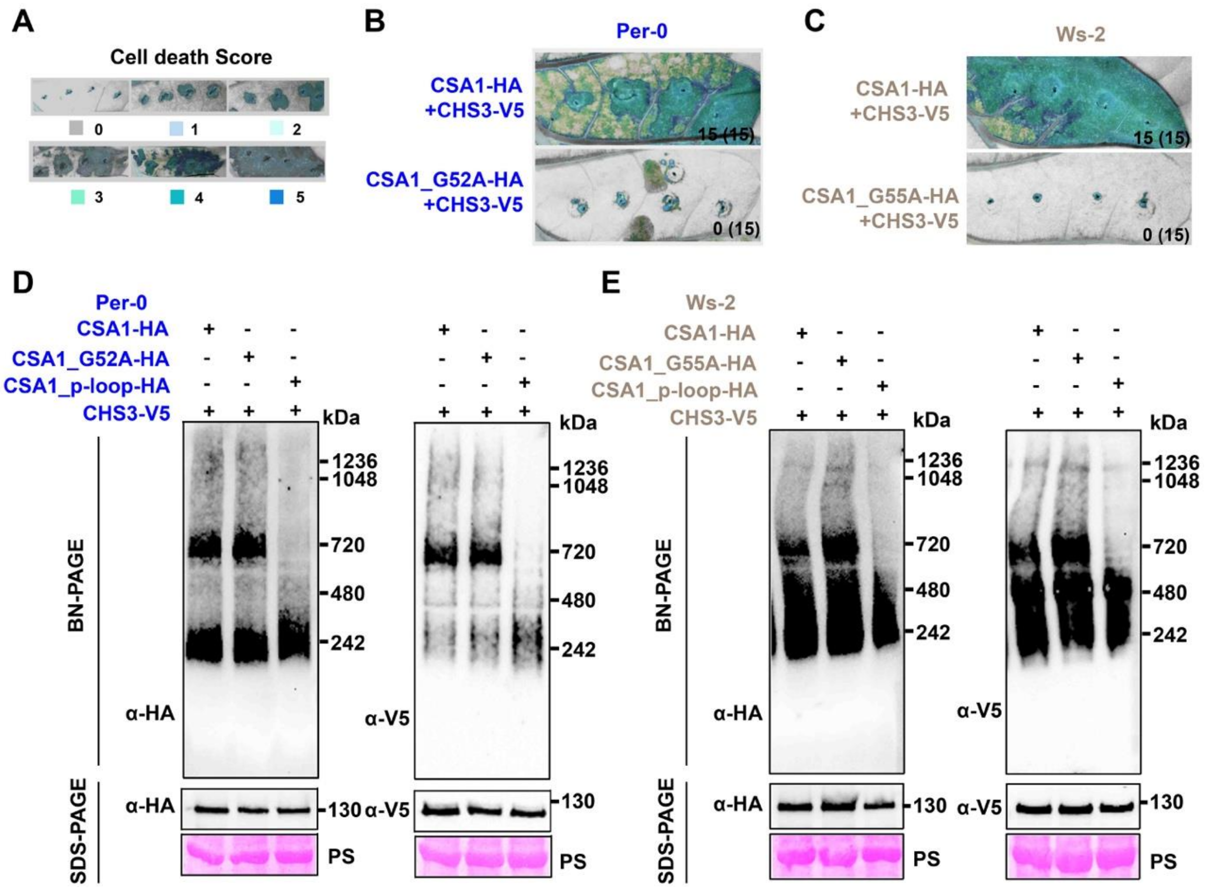

Fig. S5. The conserved glycine residue in CSA1^{TIR} BB-loop is required for cell death induction but not for oligomerization. (**A**) Representative image of cell death score used throughout to determine the extent of cell death. The same set of images were reused from Yang et al., (2022) for internal consistency. (**B** and **C**) In planta (*N. tabacum*) phenotypes. The G-to-A mutation in CSA1^{TIR} BB-loop abolishes the CHS3-CSA1 pair-mediated cell death in clade 2 (B) and clade 3 (C). Clade 2 accession and proteins are in blue and clade 3 are in grey. The numbers indicate the numbers of leaves showing cell death out of the total number of leaves infiltrated. (**D** and **E**) The conserved glycine residue in CSA1^{TIR} BB-loop is dispensable for the oligomerization of CHS3-CSA1 pair from either clade 2 (D) or clade 3 (E). CSA1_p-loop is a negative control. PS, ponceau stain indicates protein loading.

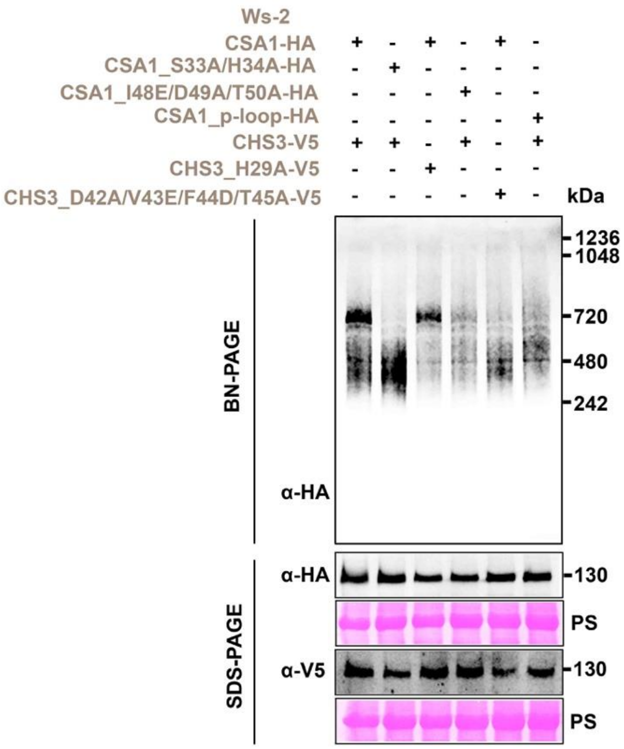

Fig. S6. The oligomerization of clade 3 Ws-2 CHS3-CSA1 pair. Both AE interface and BB-loop in TIR domains of clade 3 Ws-2 CHS3 and CSA1 affect the oligomerization. PS, ponceau stain indicates protein loading.

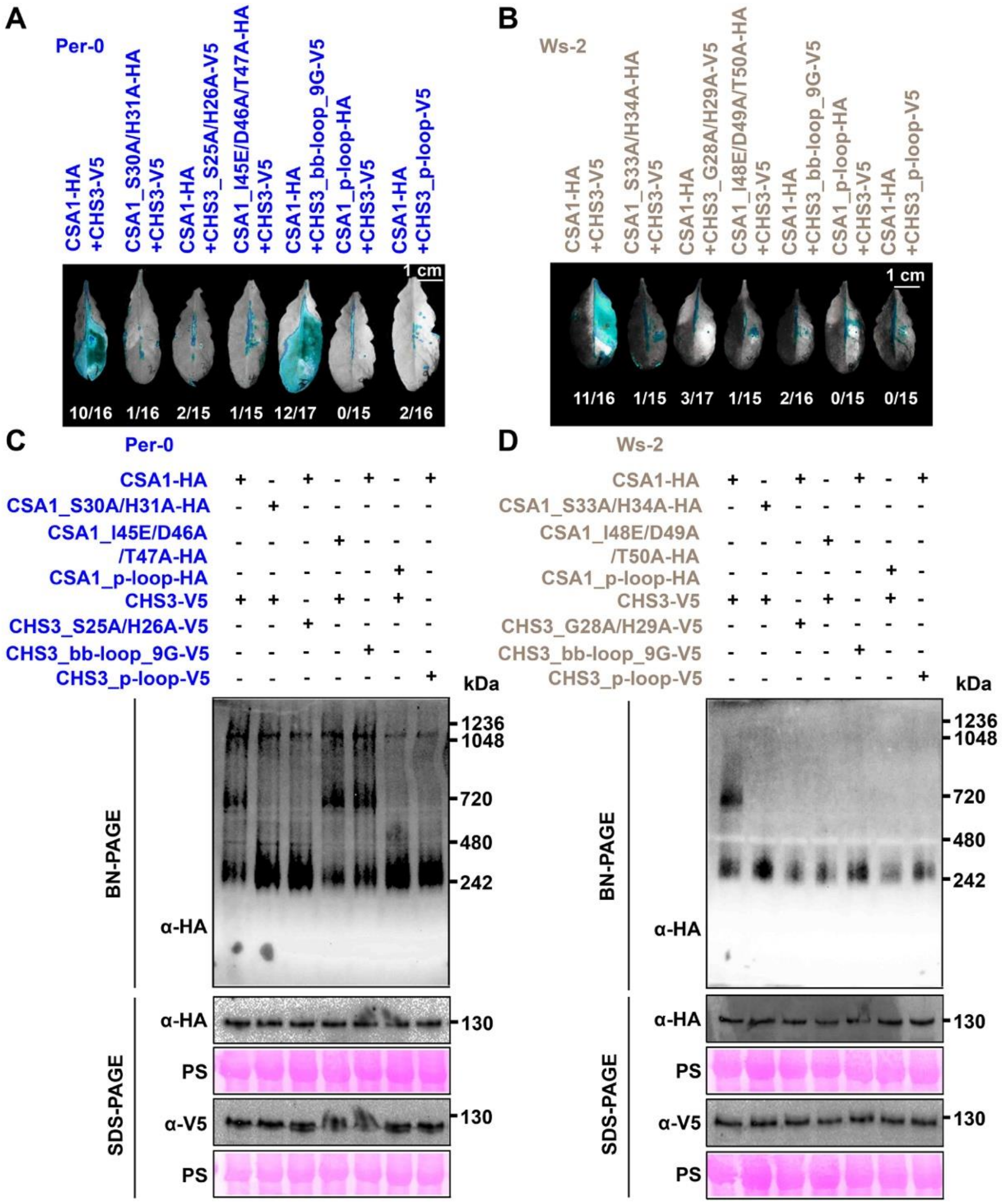

Fig. S7. Differential requirements of TIR domain for function and oligomerization between clade 2 and clade 3 pairs are maintained in *Arabidopsis*. (**A** and **B**) In planta (*Arabidopsis*) phenotypes. The intact P-loops and AE interface in TIR domains of both CSA1 and CHS3 from clade 2 Per-0 (A) or clade 3 Ws-2 (B) are required for cell death induction (Cyan leaf zones indicate the cell death). For clade 2 Per-0, the CSA1 TIR domain BB-loop is indispensable for cell death induction (A). However, for clade 3 Ws-2, the TIR domain BB-loops of both CSA1 and CHS3 are necessary for function (B). Clade 2 accession and proteins are in blue and clade 3 are in grey. The numbers indicate the number of leaves showing cell death out of the total number of leaves infiltrated. (**C** and **D**) Conserved and clade-specific TIR domain features are required for oligomerization of clade 2 and clade 3 CHS3-

CSA1 pairs. Intact P-loops and AE interface in TIR domains of both CSA1 and CHS3 are required for oligomerization in clade 2 Per-0 (C) and clade 3 Ws-2 (D). The BB-loop in the TIR domains of the clade 2 Per-0 CSA1 and CHS3 pair is dispensable for oligomerization but is indispensable for oligomerization of the clade 3 Ws-2 pair. PS, ponceau stain indicates protein loading.

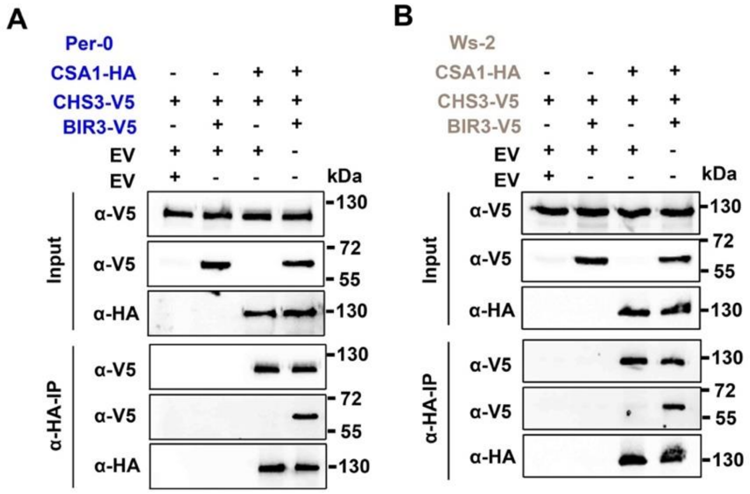

Fig. S8. BIR3 does not alter hetero-dimer formation of CHS3-CSA1 TNL pairs. (**A** and **B**) Co-IP assays showing BIR3 cannot affect the protein interaction of CHS3 and CSA1 in both clade 2 (A) and clade 3 (B). EV, empty vector. Clade 2 accession and proteins are in blue and clade 3 are in grey.

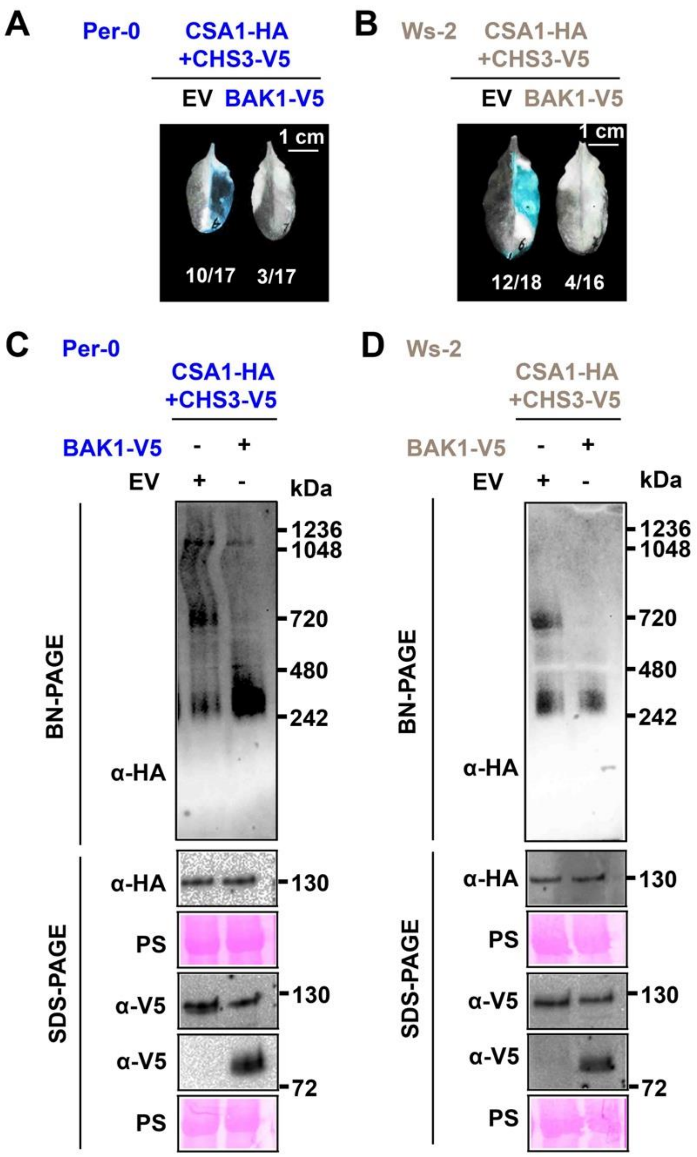

Fig. 9. BAK1 suppress the oligomerization of the CHS3-CSA1 TNL pair in *Arabidopsis*. (**A** and **B**) In planta (*Arabidopsis*) phenotypes. Co-expression of BAK1 could inhibit cell death phenotype (Cyan leaf zones indicate the cell death) induced by TNL CHS3-CSA1 pair from clade 2 Per-0 (A) or clade 3 Ws-2 (B) in *Arabidopsis*. EV, empty vector. The numbers indicate the numbers of leaves displaying cell death out of the total number of leaves infiltrated. (**C** and **D**) BN-PAGE assays show that BAK1 suppresses the oligomerization of the CHS3-CSA1 pair from clade 2 Per-0 (C) or clade 3 Ws-2 (D). EV, empty vector. PS, ponceau stain indicates protein loading. Clade 2 accession and proteins are in blue and clade 3 are in grey.

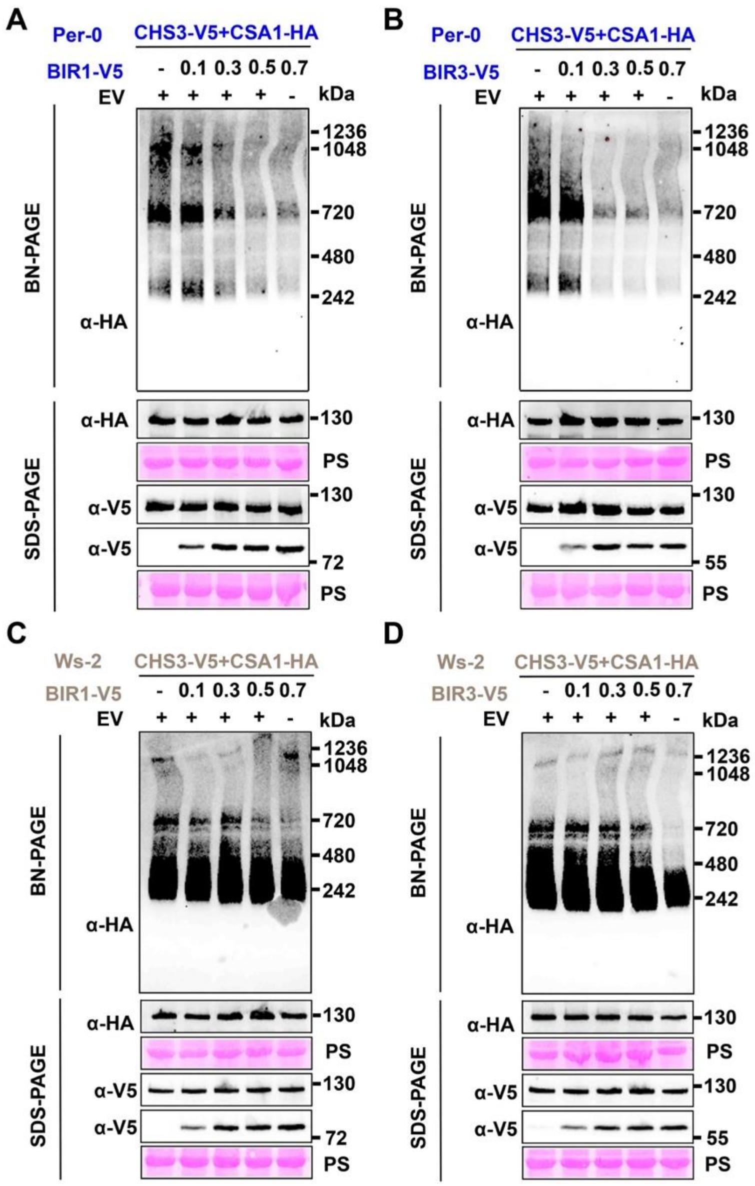

Fig. S10. The oligomerization of CHS3-CSA1 pair is inhibited by BIRs. (**A** to **D**) BIR1 [(A) and (C)] and BIR3 [(B) and (D)] suppress the hetero-oligomeric complex formation of CHS3-CSA1 pair in a dose-dependent manner in clade 2 [(A) and (B)] and clade 3 [(C) and (D)]. EV, empty vector. PS, ponceau stain indicates protein loading. Clade 2 accession and proteins are in blue and clade 3 are in grey. Values above lanes (e.g. 0.1) indicate amount of each Agrobacterium strain infiltrated, OD=600 nm.

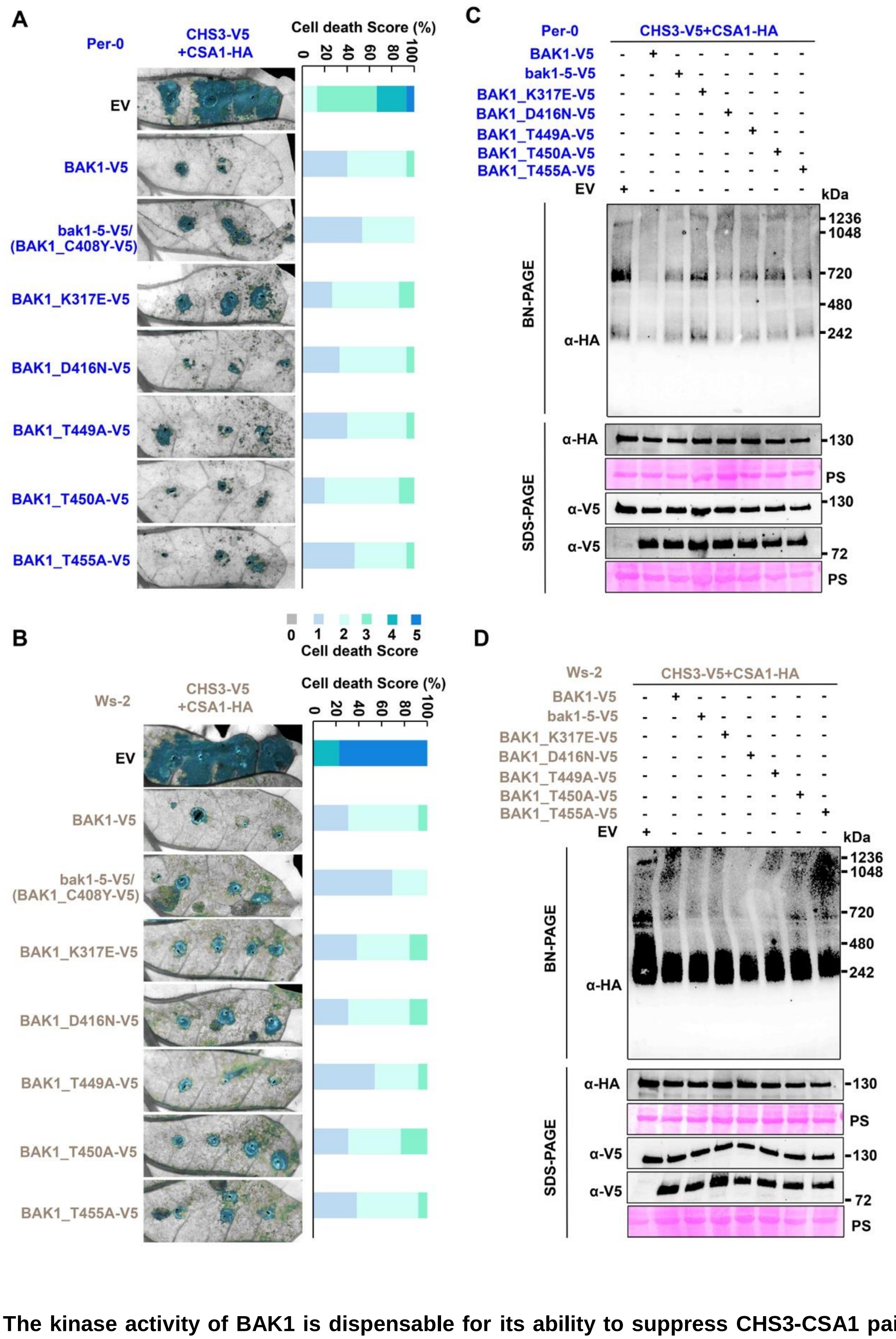

Fig. S11. The kinase activity of BAK1 is dispensable for its ability to suppress CHS3-CSA1 pair function and oligomerization. (**A** and **B**) In planta (*N. tabacum*) phenotypes (left) and corresponding percentage

representations of cell death score (right). The bak1-5 and a series of kinase-inactive BAK1 mutants are still able to inhibit the CHS3-CSA1 pair-triggered cell death in both clade 2 (A) and clade 3 (B). EV, empty vector. Stacked bars are color-coded showing the proportions (in percentage) of each cell death score (0 to 5). Fifteen (Per-0) and thirteen (Ws-2) leaves were scored for each stacked bar, respectively. (**C** and **D**) BN-PAGE assays demonstrating that oligomerization of CHS3-CSA1 pair from either clade 2 Per-0 (C) or clade 3 Ws-2 (D) is suppressed by wild-type BAK1, bak1-5 and various loss-of-kinase activity BAK1 mutants. EV, empty vector. PS, ponceau stain indicates protein loading. Clade 2 accession and proteins are in blue and clade 3 are in grey.

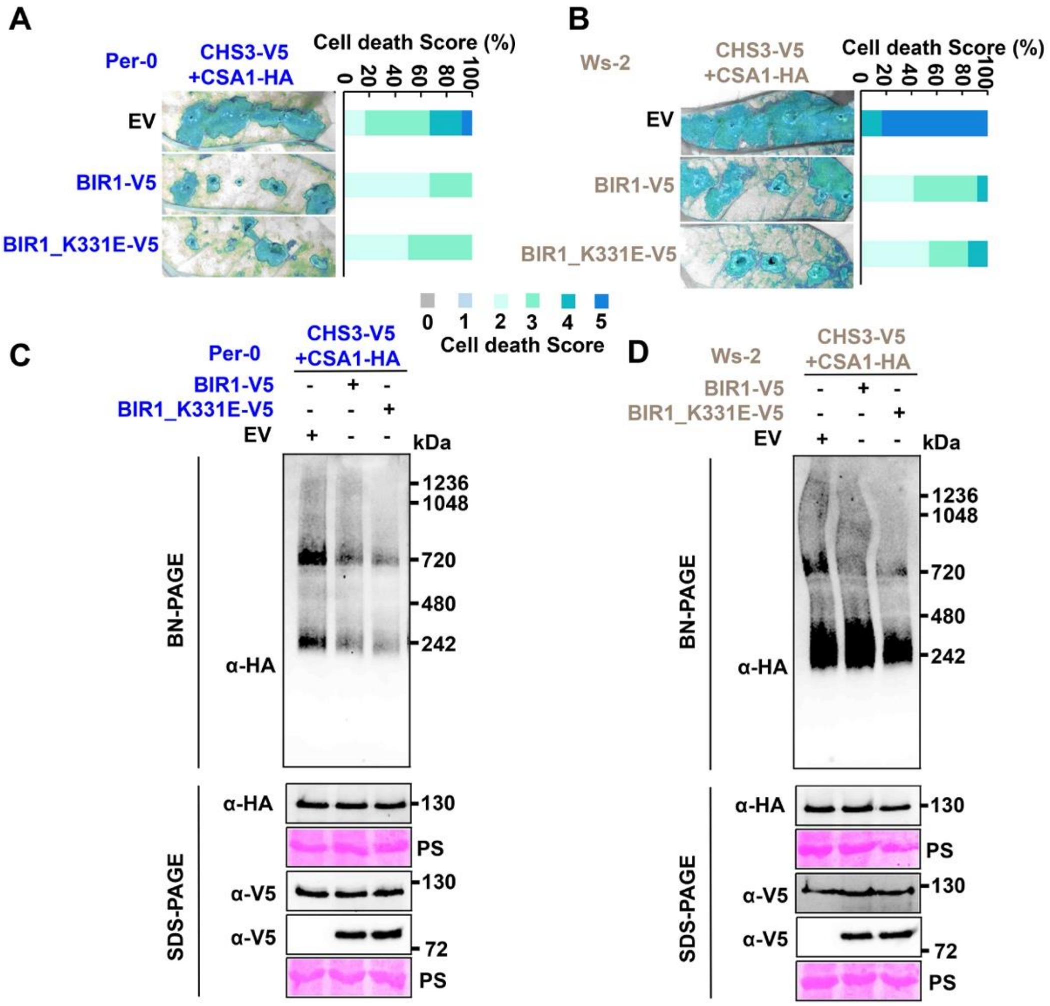

Fig. S12. The kinase activity of BIR1 is not necessary to inhibit CHS3-CSA1 pair-mediated cell death and oligomerization. (**A** and **B**) In planta (*N. tabacum*) phenotypes (left) and corresponding percentage representations of cell death score (right). the BIR1_K331E mutant, which dramatically reduced the kinase activity of BIR1, is still able to inhibit the CHS3-CSA1 pair-mediated cell death in both clade 2 (A) and clade 3 (B). EV, empty vector. Stacked bars are color-coded showing the proportions (in percentage) of each cell death score (0 to 5). Twelve leaves were scored for each stacked bar. (**C** and **D**) BN-PAGE assays showing the kinase activity of BIR1 is not required for suppression of CHS3-CSA1 pair oligomerization in clade 2 (C) and clade 3 (D). EV, empty vector. PS, ponceau stain indicates protein loading. Clade 2 accession and proteins are in blue and clade 3 are in grey.

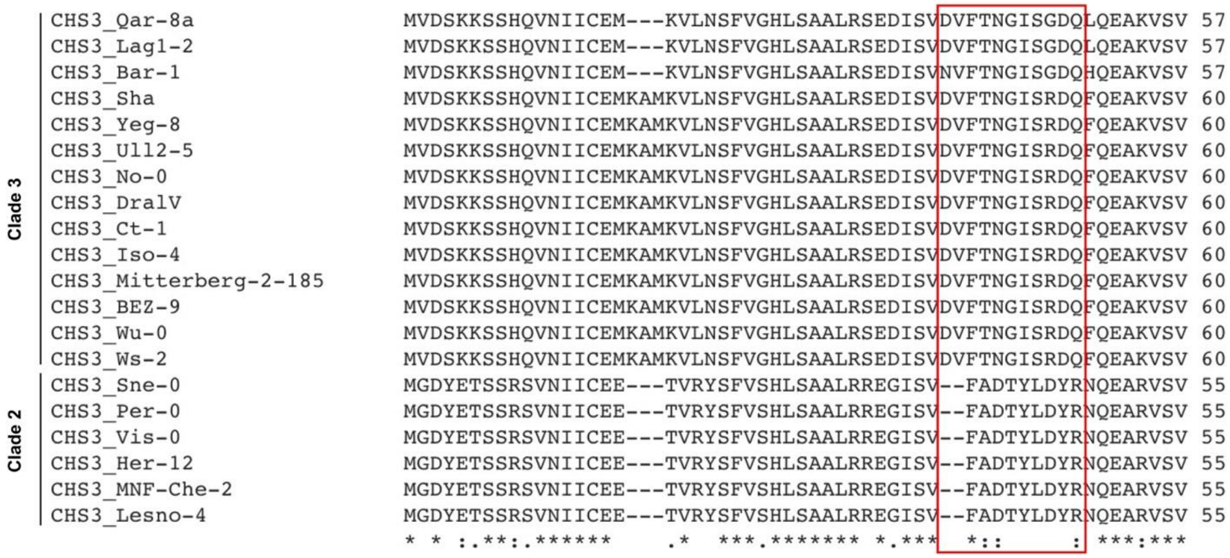

Fig. S13. Protein sequence alignment of CHS3 from clade 2 and clade 3. the CHS3^{TIR} BB-loop is only conserved within but not between clades. The red box indicates the BB-loop.

Table S1: Gene mutants used in this study

Mutants	References and rationale
**NADase catalytic dead mutants**	The number represents relevant papers cited as the rationale for each much allele.
CSA1_E85A (Per-0)	
CSA1_E88A (Ws-2)	*21-23, 43*
**P-loop dead mutants (nucleotide-binding site)**	
CSA1_p-loop (G236A/K237A/T238A) (Per-0)	
CSA1_p-loop (G238A/K239A/S240A) (Ws-2)	
CHS3_p-loop (G171A/K172A/T173A) (Per-0)	
CHS3_p-loop (G174A/K175A/T176A) (Ws-2)	*18-26, 43*
**AE interface in TIR domain mutants (TIR domain interaction)**	
CSA1_S30A/H31A (Per-0)	
CSA1_S33A/H34A (Ws-2)	
CHS3_S25A/H26A (Per-0)	
CHS3_H29A (Ws-2)	
CHS3_G28A/H29A (Ws-2)	*21, 22, 45*
**BB-loop in TIR domain mutants (TIR domain interaction)**	
CSA1_I45E/D46A/T47A (Per-0)	These positions were chosen based on protein alignment (*21, 22*) (fig. S4C). The mutation of DT to AA is based on the reference *50*, and the mutation of I to E is based on change of charge and side chain of amino acid.
CSA1_I48E/D49A/T50A (Ws-2)	
CSA1_G52A (Per-0)	
CSA1_G55A (Per-0)	*22*
CHS3_F39D/A40E/D41A/T42A (Per-0)	These positions were chosen based on protein alignment (*21, 22*) (fig. S4C). The mutation we generated is based on change of charge and side chain of each amino acid.
CHS3_D42A/V43E/F44D/T45A (Ws-2)	
CHS3_F39G/A40G/D41G/T42G (Per-0)	This position we chose to make mutation is based on the protein alignment (*21, 22*) (fig. S4C), and the mutation we made is based on the reference *51*.
CHS3_Y43G/L44G/D45G/Y46G/R47G (Per-0)	
CHS3_bb-loop_9G (Per-0)	
CHS3_bb-loop_9G (Ws-2)	
**others**	
BAK1 mutants	*43, 52, 55*
BIR1 mutant	*57*

Table S2: List of Primers used in this study

Name	Sequence (5'-3')
CSA1_Per-0 SH/AA-R1	tataGGTCTCtAGCTTTTCTTGAGaGCaGCGACAAAGTT
CSA1_Per-0 SH/AA-F2	tataGGTCTCtAGCTTAAAAAGGAATGGGATCAATGCC
CHS3_Per-0 SH/AA-R1	tataGGTCTCtGGAGAGgGCgGCGACGAAAGAGTA
CHS3_Per-0 SH/AA-F2	tataGGTCTCtCTCCGCCGCTTTACGCCGCG
CSA1_Per-0 IDT/EAA-R1	tataGGTCTCtGCCCATATCTTCATCtgCgGctTCGAAGG
CSA1_Per-0 IDT/EAA-F2	tataGGTCTCtgggcCAAGAACTAAATGTTCTGCTC
CHS3_Per-0 FADT/DEAA-R1	tataGGTCTCtCGAGATAtgCaGccTcaTCCACAGAGAT
CHS3_Per-0 FADT/DEAA-F2	tataGGTCTCtCTCGATTATCGAAATCAGGAAGCTAGGG
CHS3_Per-0 FADT/GGGG-R1	tataGGTCTCtGAGATAtCCaCccCcaCCCACAGAGATCCCTTCGCG
CHS3_Per-0 FADT/GGGG-F2	tataGGTCTCtTctcgattatcgaaatcaggaagctagg
CHS3_Per-0 YLDYR/GGGGG-R1	tataGGTCTCtTCCTGATTtcCaCCaCcgCCaCCTGTATCCGCAAACACAGAGATCCC
CHS3_Per-0 YLDYR/GGGGG-F2	tataGGTCTCtaggaagctagggtttctgtggtag
CHS3_Per-0 bb-loop-9G-R1	tataGGTCTCtTCCTGATTtcCaCCaCcgCCaCCtCCaCccCcaCcCACAGAGATCCC
CSA1_Per-0 G52A-R1	tataGGTCTCtGCAGAACATTTAGTTCTTGgGcCATATC
CSA1_Per-0 G52A-F2	tataGGTCTCtCTGCTCAAAAGAATCGAGGGTTCG
CSA1_Ws-2 SH/AA-R1	tataGGTCTCtCGAGaGCaGCAACAAAGTTGTAGCGC
CSA1_Ws-2 SH/AA-F2	tataGGTCTCtCTCGAGAAGGAATTAAAAAGGAATGGG
CHS3_Ws-2 H/A-R1	tataGGTCTCtGCGGATAGgGCACCGACGAA
CHS3_Ws-2 H/A-F2	tataGGTCTCtCCGCCGCTTTACGTAGCGAA
CHS3_Ws-2 GH/AA-R1	tataGGTCTCtCGGATAGgGCaGcGACGAAGGA
CHS3_Ws-2 GH/AA-F2	tataGGTCTCtTCCGCCGCTTTACGTAGCGA
CSA1_Ws-2 IDT/EAA-R1	tataGGTCTCtGCCCATATCTTCATCtgCgGctTCGAAGG
CSA1_Ws-2 IDT/EAA-F2	tataGGTCTCtGGGCCAAGAGTTGAATGTTCTGC
CHS3_Ws-2 DVFT/AEDA-R1-2	tataGGTCTCtCACGAGAAATTCCATTtgCaTCcTcaGcGAC
CHS3_Ws-2 DVFT/AEDA-F2-2	tataGGTCTCtCGTGATCAGTTTCAGGAAGCTAAGGT
CHS3_Ws-2 bb-loop-9G-R1	tataGGTCTCtcCCaCcacCaCCaCCtccaCCtCCaCCcacatcgacagagatgtcttcgct
CHS3_Ws-2 bb-loop-9G-F2	tataGGTCTCttGGgTTTCAGGAAGCTAAGGTTTCGGTG
CSA1_Ws-2 G55A-R1	tataGGTCTCtACTCTTGgGcCATATCTTCATCTGTG
CSA1_Ws-2 G55A-F2	tataGGTCTCtGAGTTGAATGTTCTGCTCAAGAGAATCG
